# Identification of potential metabolic biomarkers of cerebrospinal fluids that differentiate tuberculous meningitis from other types of meningitis by a metabolomics study

**DOI:** 10.18632/oncotarget.21942

**Published:** 2017-10-19

**Authors:** Yi-Ning Dai, Hai-Jun Huang, Wen-Yuan Song, Yong-Xi Tong, Dan-Hong Yang, Ming-Shan Wang, Yi-Cheng Huang, Mei-Juan Chen, Jia-Jie Zhang, Ze-Ze Ren, Wei Zheng, Hong-Ying Pan

**Affiliations:** ^1^ Department of Infectious Diseases, Zhejiang Provincial People’s Hospital, People’s Hospital of Hangzhou Medical College, Hangzhou, Zhejiang, China; ^2^ Department of Infectious Diseases, The Second Affiliated Hospital of Zhejiang Chinese Medicinal University, Hangzhou, Zhejiang, China

**Keywords:** tuberculous meningitis, viral meningitis, bacterial meningitis, cryptococcal meningitis, metabolomics

## Abstract

Tuberculous meningitis (TBM) is caused by tuberculosis infection of of the meninges, which are the membrane systems that encircle the brain, with a high morbidity and mortality rate. It is challenging to diagnose TBM among other types of meningitis, such as viral meningitis, bacterial meningitis and cryptococcal meningitis. We aimed to identify metabolites that are differentially expressed between TBM and the other types of meningitis by a global metabolomics analysis. The cerebrospinal fluids (CSF) from 50 patients with TBM, 17 with viral meningitis, 17 with bacterial meningitis, and 16 with cryptococcal meningitis were analyzed using ultra high performance liquid chromatography coupled with quadrupole time of flight mass spectrometry (UHPLC-QTOF-MS). A total of 1161 and 512 features were determined in positive and negative electrospray ionization mode, respectively. A clear separation between TBM and viral, bacterial or cryptococcal meningitis was achieved by orthogonal projections to latent structures-discriminate analysis (OPLS-DA) analysis. Potential metabolic markers and related pathways were identified, which were mainly involved in the metabolism of amino acid, lipids and nucleosides. In summary, differential metabolic profiles of the CSF exist between TBM and other types of meningitis, and potential metabolic biomarkers were identified to differentiate TBM from other types of meningitis.

## INTRODUCTION

Tuberculosis (TB) remains a major global health burden. There were approximately 11 million prevalent cases, and an estimated 9 million incident cases occurred all over the world in 2013 [1]. In 2012, TB killed 1.3 million people worldwide [2]. Tuberculous meningitis (TBM) is Mycobacterium tuberculosis infection of the meninges, that is, the membranes which envelop the central nervous system (CNS). It is the most severe form of tuberculosis infection in the CNS, leading to a fatal outcome and permanent sequelae [3–5]. It is widely accepted that TBM carries a high morbidity and mortality rate [6–8]. Moreover, neurological deficits such as hemiplegia, quadriplegia, seizures, cognitive impairment and cranial nerve palsy have been frequently reported in TBM survivors [5].

Previous studies have demonstrated that early diagnosis and in-time treatment of TBM is critical in reducing complications and mortality rates [9–11]. However, there is no universally accepted method for differentiating TBM diagnosis from other meningitis, such as viral meningitis, cryptococcal meningitis and bacterial meningitis, thus resulting in delayed intervention, and a higher morbidity and mortality rate. Hence, the development of a more accurate diagnosis based on molecular biology for TBM is necessary.

Metabolomics is the quantitative analysis of a comprehensive profile of metabolites in biological samples, which provides new insight into the metabolic pathway of disease and identifies potential disease markers [12]. It is well known that TB infection can alter metabolic processes. As TB transgresses the blood-brain barrier (BBB) and invades the CNS, the cerebrospinal fluid (CSF) presents with a predominance of lymphocytes, increased protein content and low glucose concentration. Previous studies using nuclear magnetic resonance (NMR)-based metabolomics approach have identified elevated levels of lactic acid in the CSF of TBM infants and children compared to non-meningitis controls [13, 14]. Until now, data regarding to the systematic characteristics of CSF metabolome in TBM and other meningitis is very scarcity. In this study, we used the ultra high performance liquid chromatography coupled with quadrupole time of flight mass spectrometry (UHPLC-QTOF-MS) to analyze CSF collected from patients with TBM, viral meningitis, cryptococcal meningitis and bacterial meningitis, in the purpose of discovering potential biomarkers for the differential diagnosis of meningitis, and providing new insights into the pathophysiology of TBM.

## RESULTS

### Baseline characteristics and CSF metabolic profiles

50 patients diagnosed as TBM, 17 subjects with viral meningitis, 17 with bacterial meningitis, and 16 with cryptococcal meningitis were included in this study. The age (average ± standard deviation) of patients in TBM, viral meningitis, bacterial meningitis and cryptococcal meningitis was 47.58 ± 17.52, 38.24 ± 20.22, 44.59 ± 20.16, 46.94 ± 10.00, respectively. Two-tailed t test witnessed no significant difference among the four groups. The participants included 64 males and 36 females. No difference of gender distribution was observed in each group of comparison.

A total of 100 CSF samples were analyzed by UHPLC-QTOF-MS non-targeting profiling method. Instrument performance was constantly monitored by 28 QC samples. After the de-noising procedure and data normalization, 1161 (positive) and 512 (negative) features were identified and applied for further investigation.

### Multivariate data analyses

**Figure 1:**
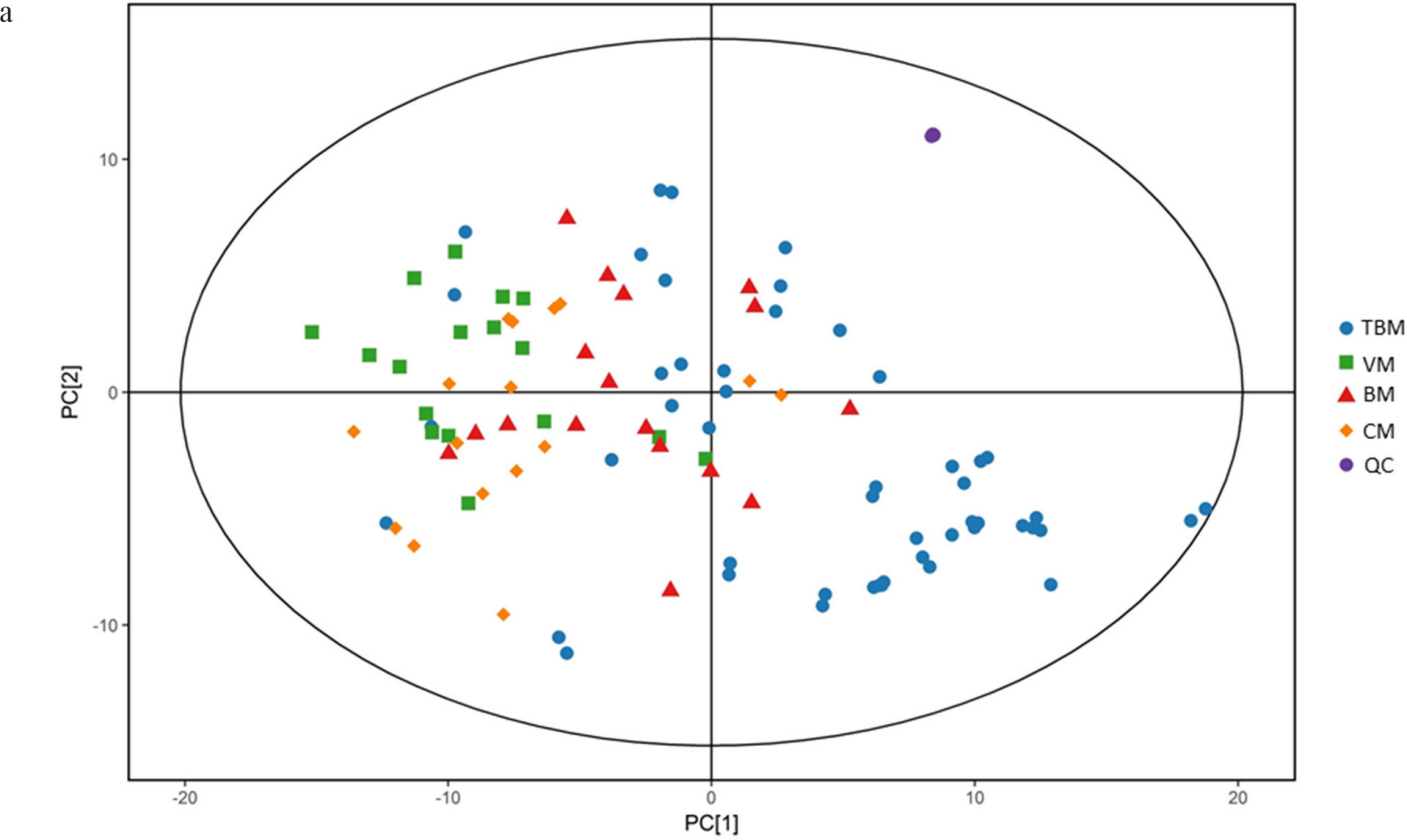

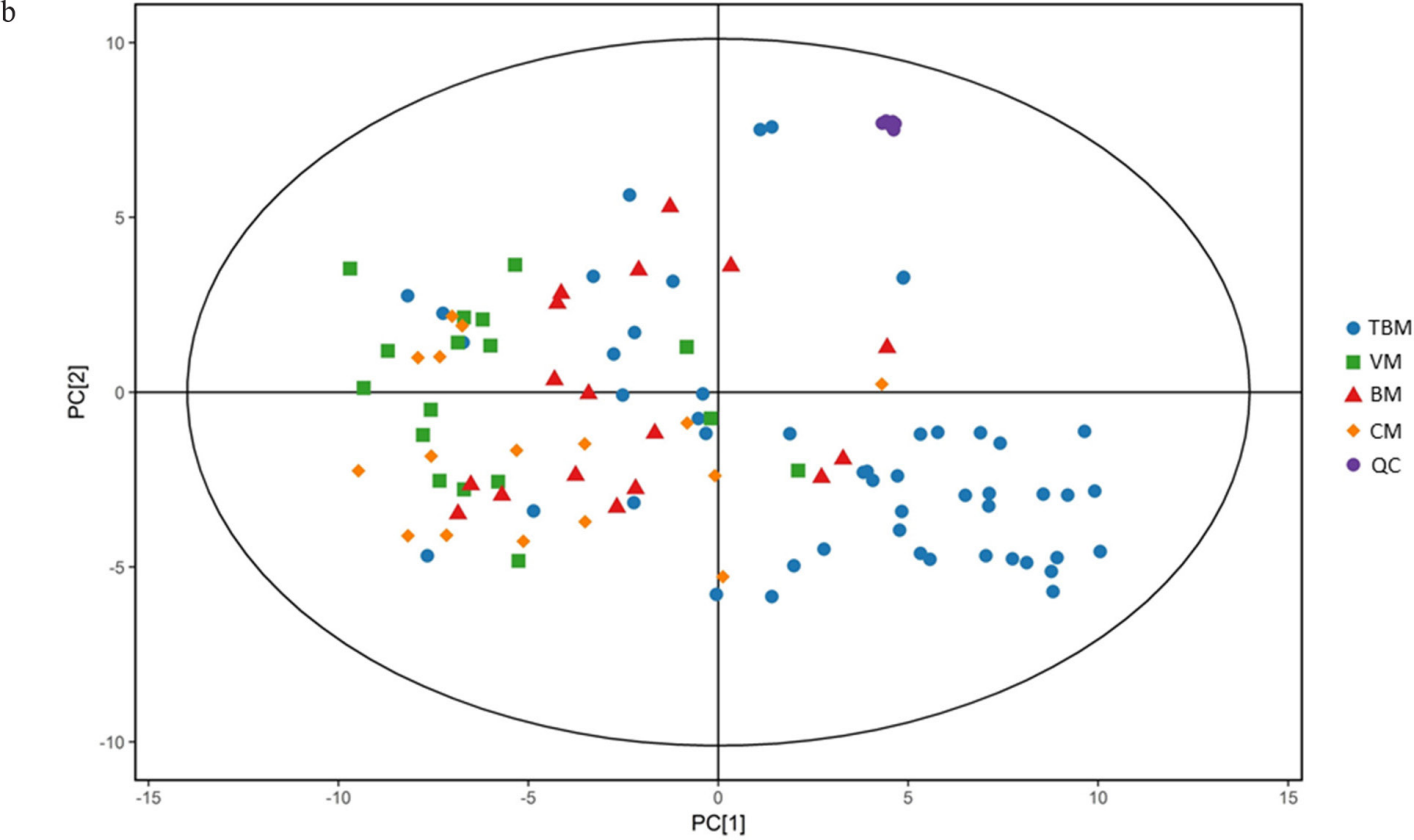

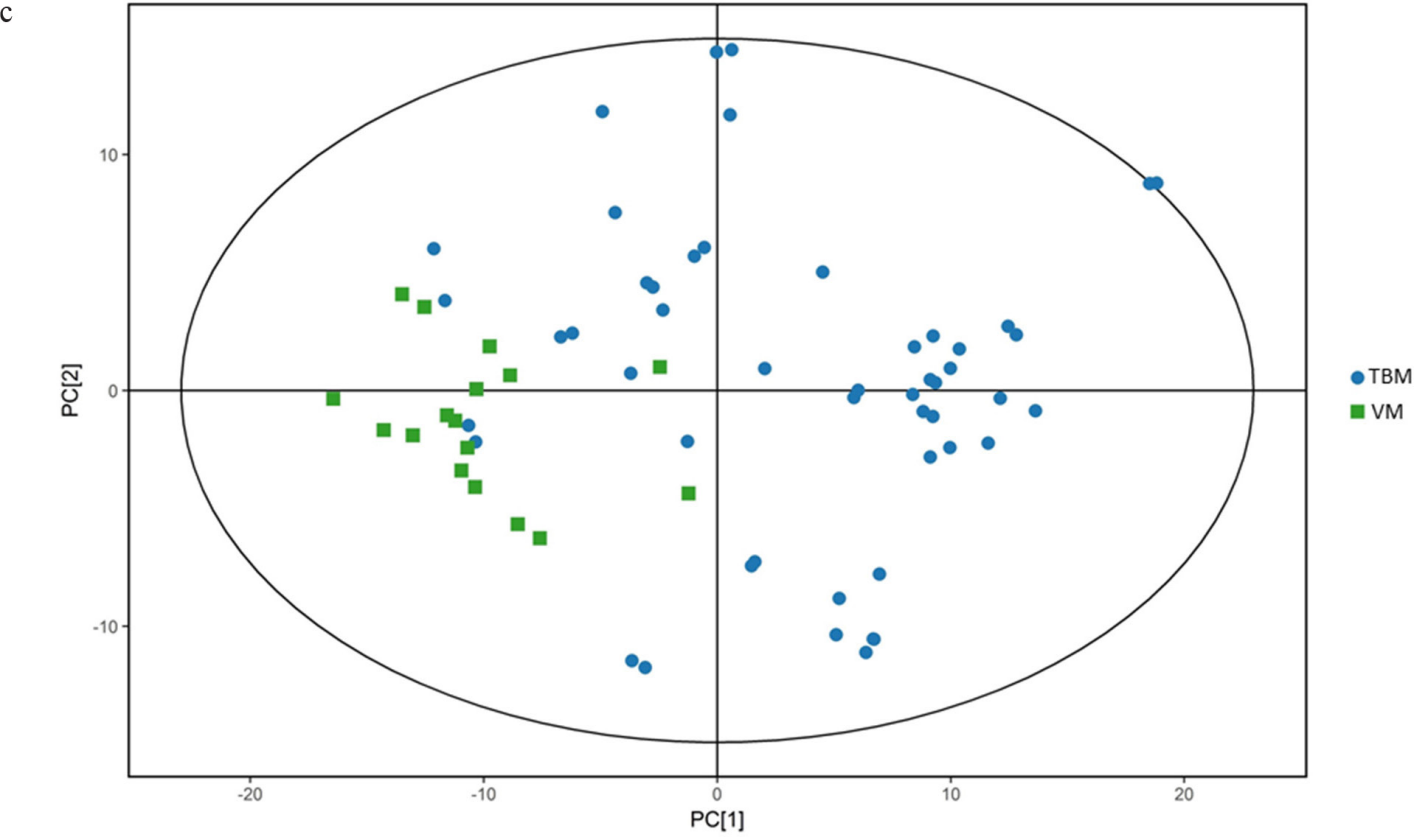

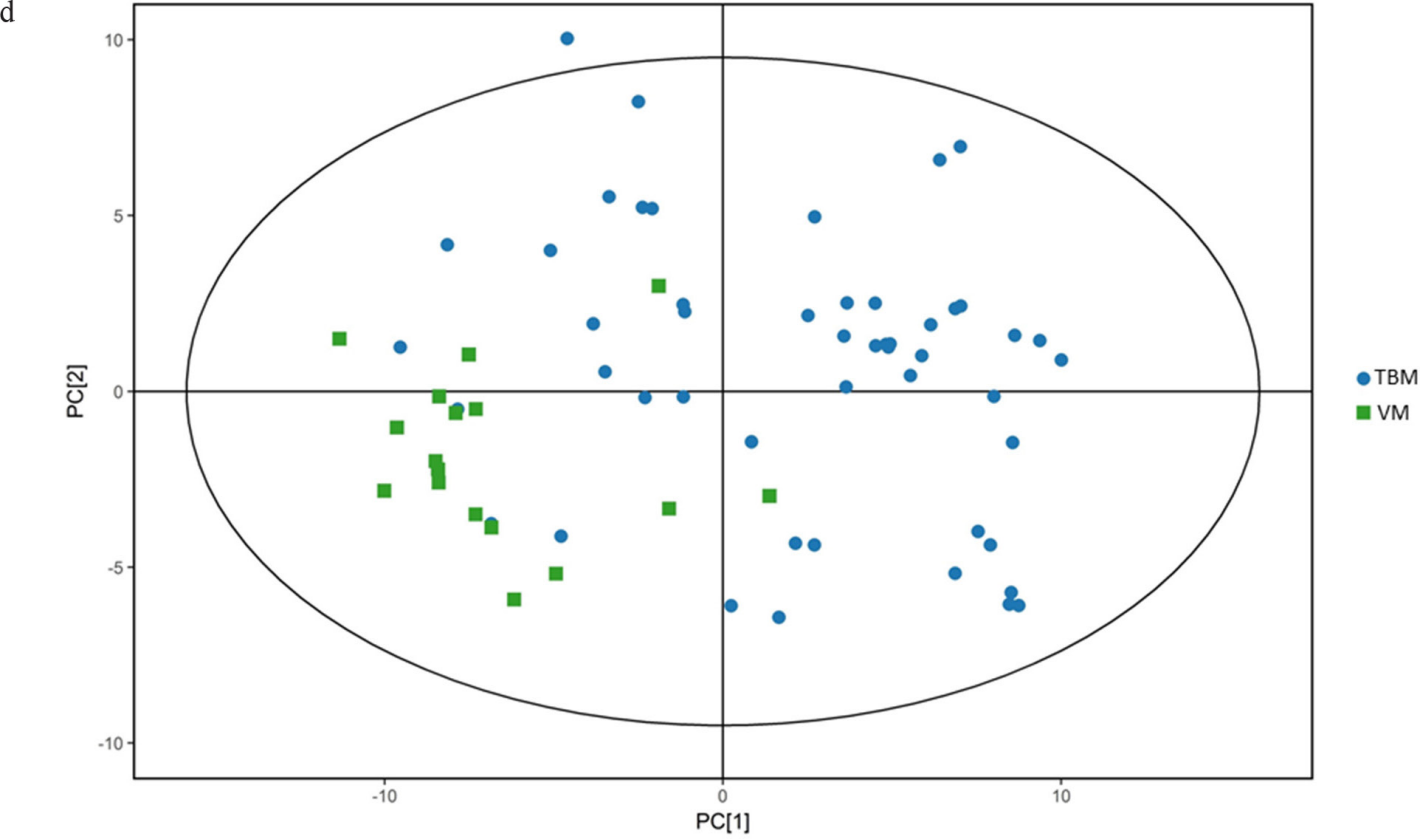

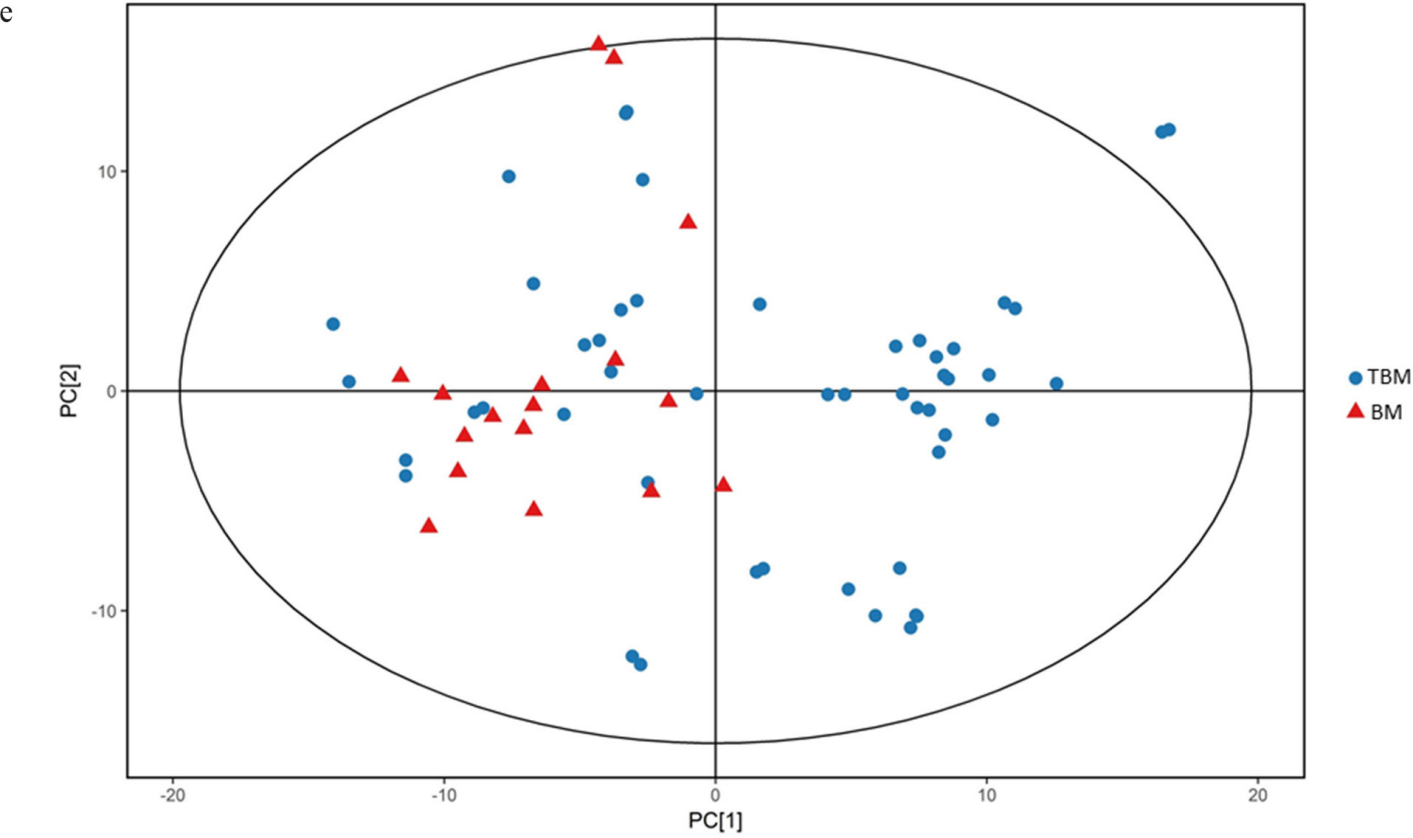

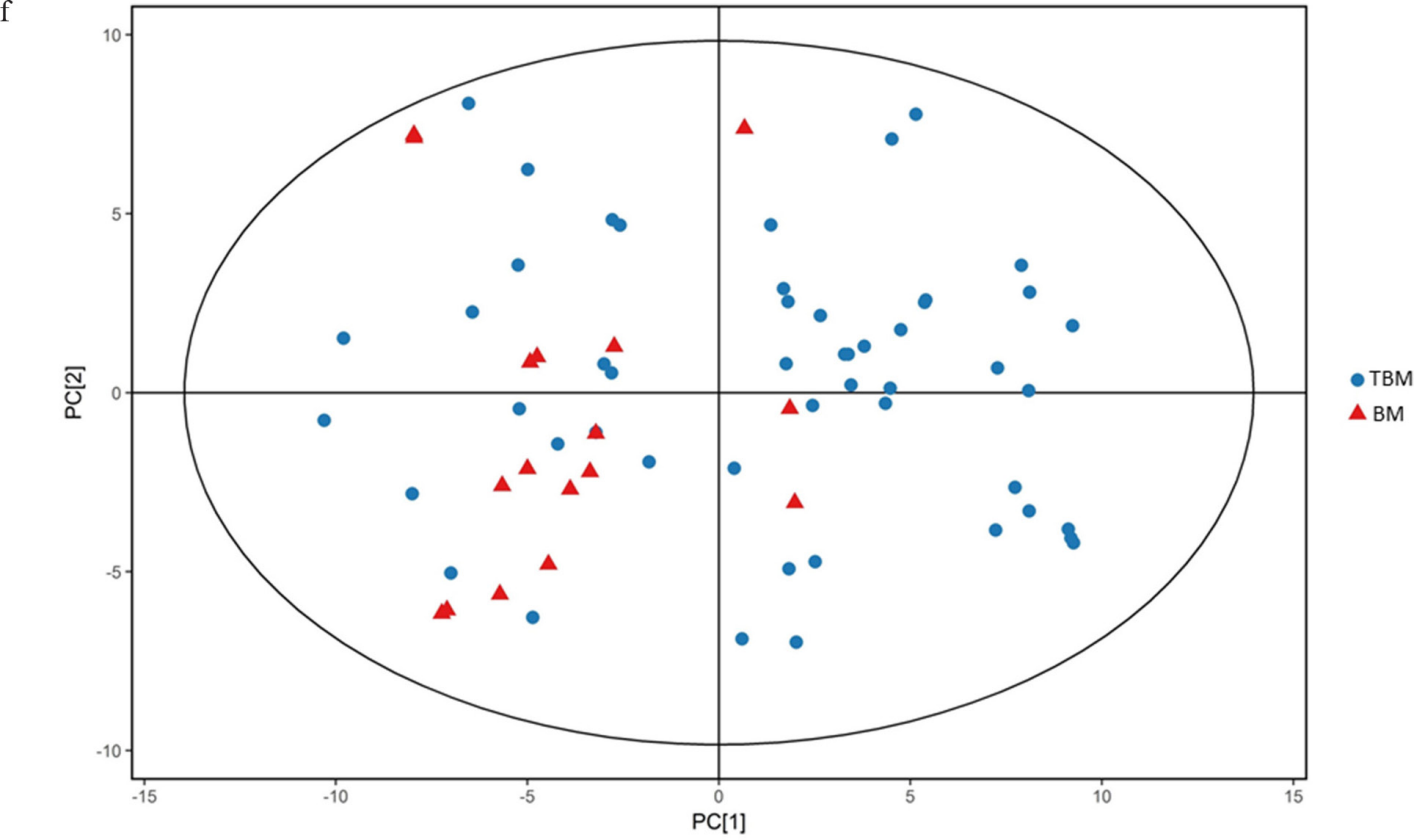

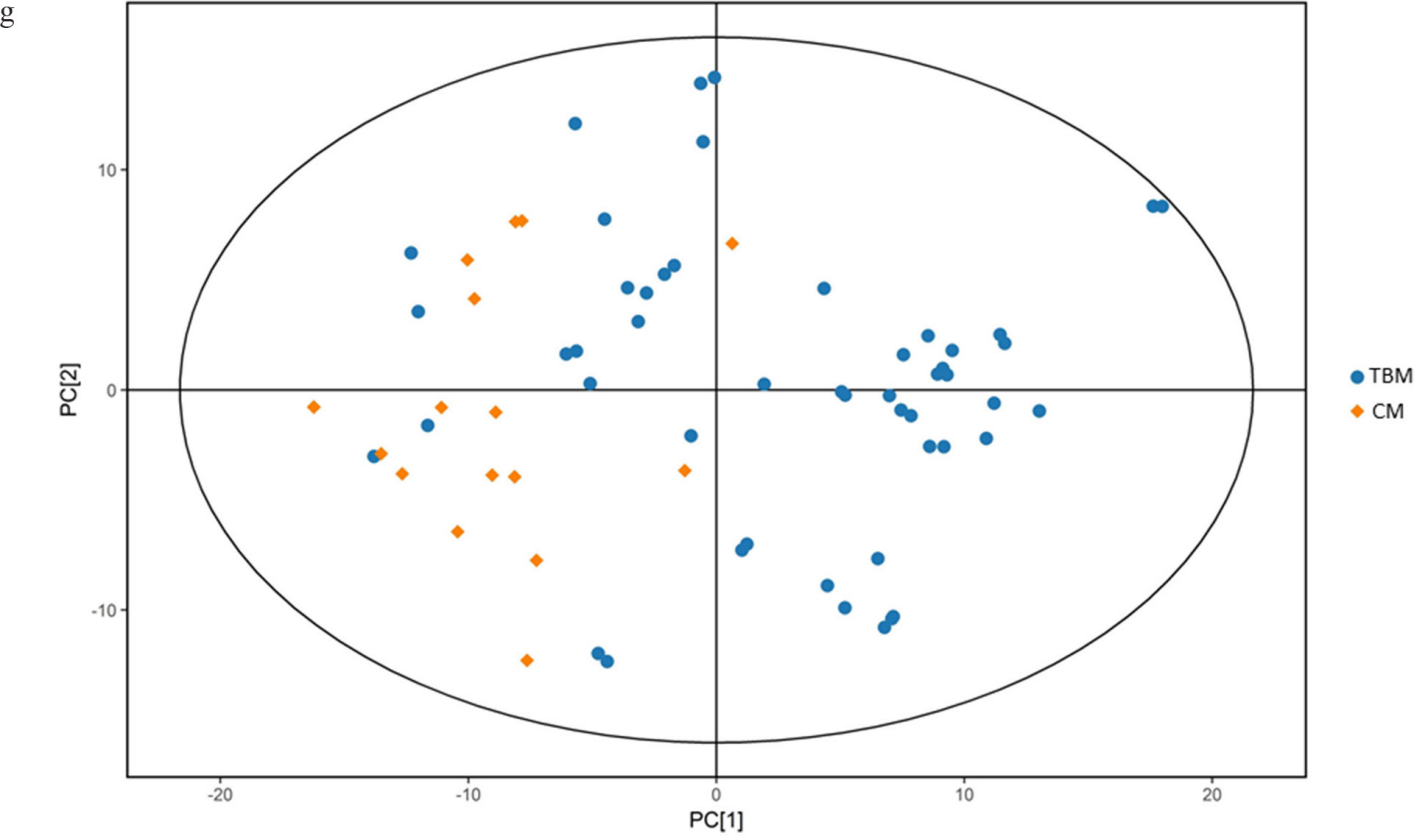

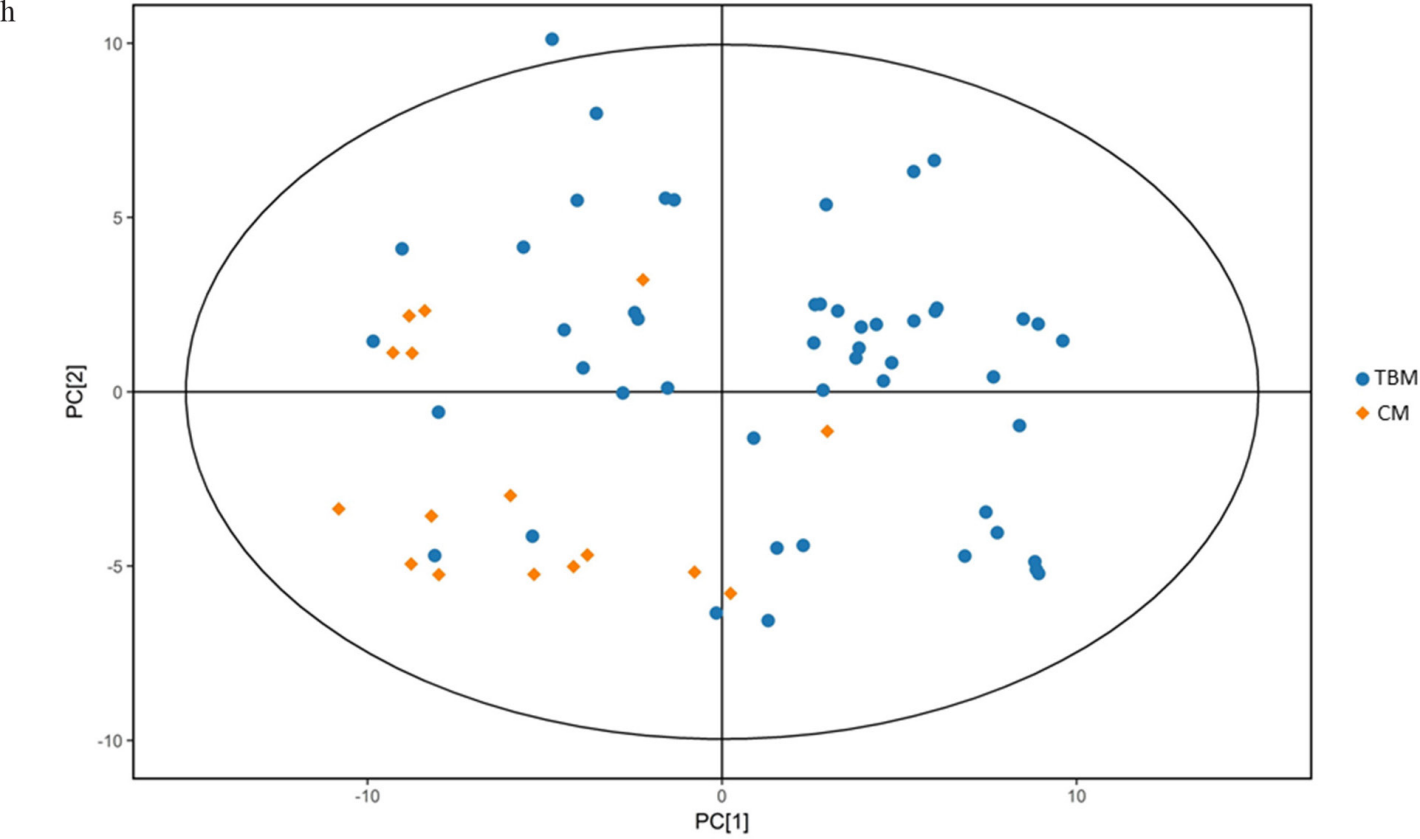
Scores scatter plots of principal component analyses (PCA) of cerebrospinal fluids (CSF) from tuberculous meningitis (TBM), viral meningitis (VM), bacterial meningitis (BM) and cryptococcal meningitis (CM) **(a)** All 100 CSF samples along with the quality control (QC) samples in positive electrospray ionization (ESI) mode. **(b)** All 100 CSF samples along with the quality control (QC) samples in negative ESI mode. **(c)** **CSF samples from TBM and VM in positive ESI mode.** **(d)** CSF samples from TBM and VM in negative ESI mode. **(e)** CSF samples from TBM and BM in positive ESI mode. **(f)** CSF samples from TBM and BM in negative ESI mode. **(g)** CSF samples from TBM and CM in positive ESI mode. **(h)** CSF samples from TBM and CM in negative ESI mode. No discernible separation among the four groups was observed by PCA analysis.

**Figure 2:**
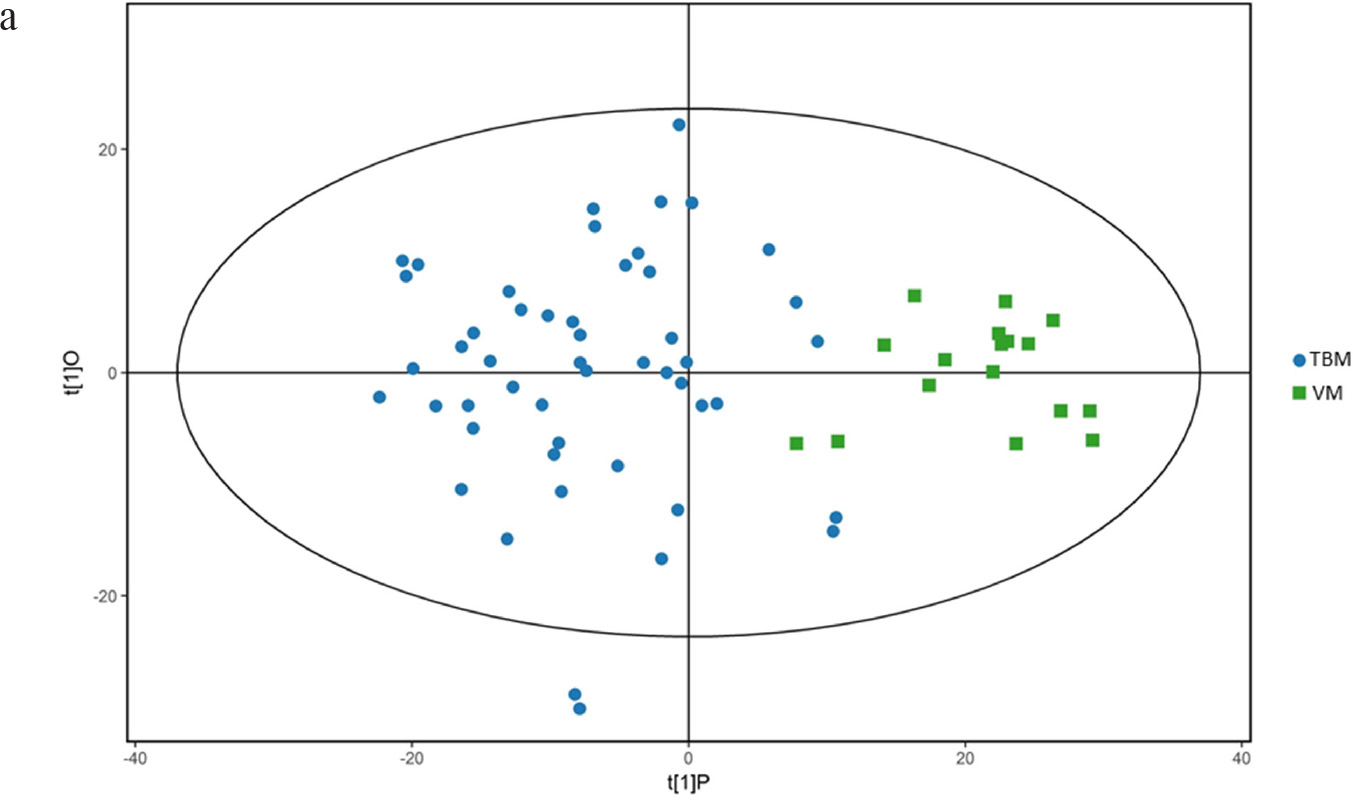

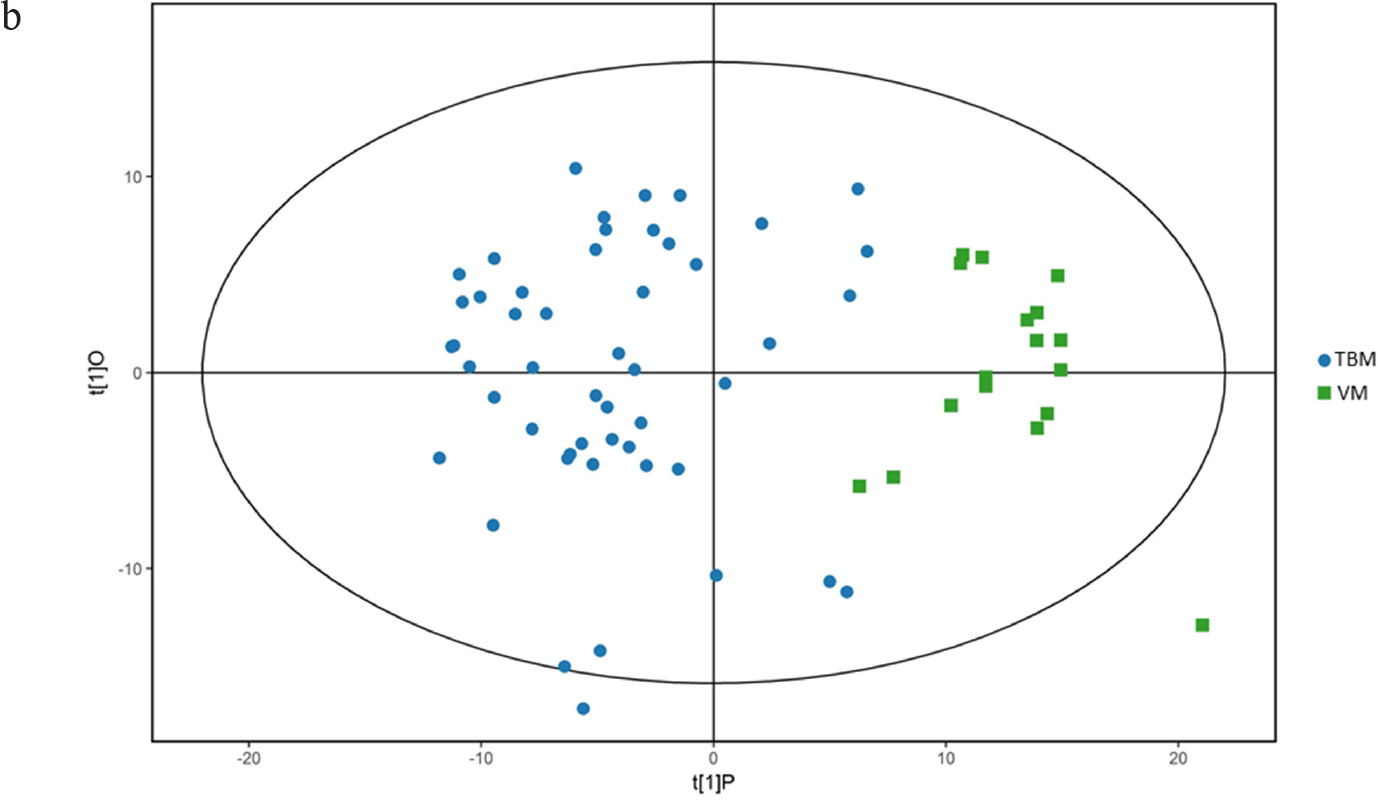

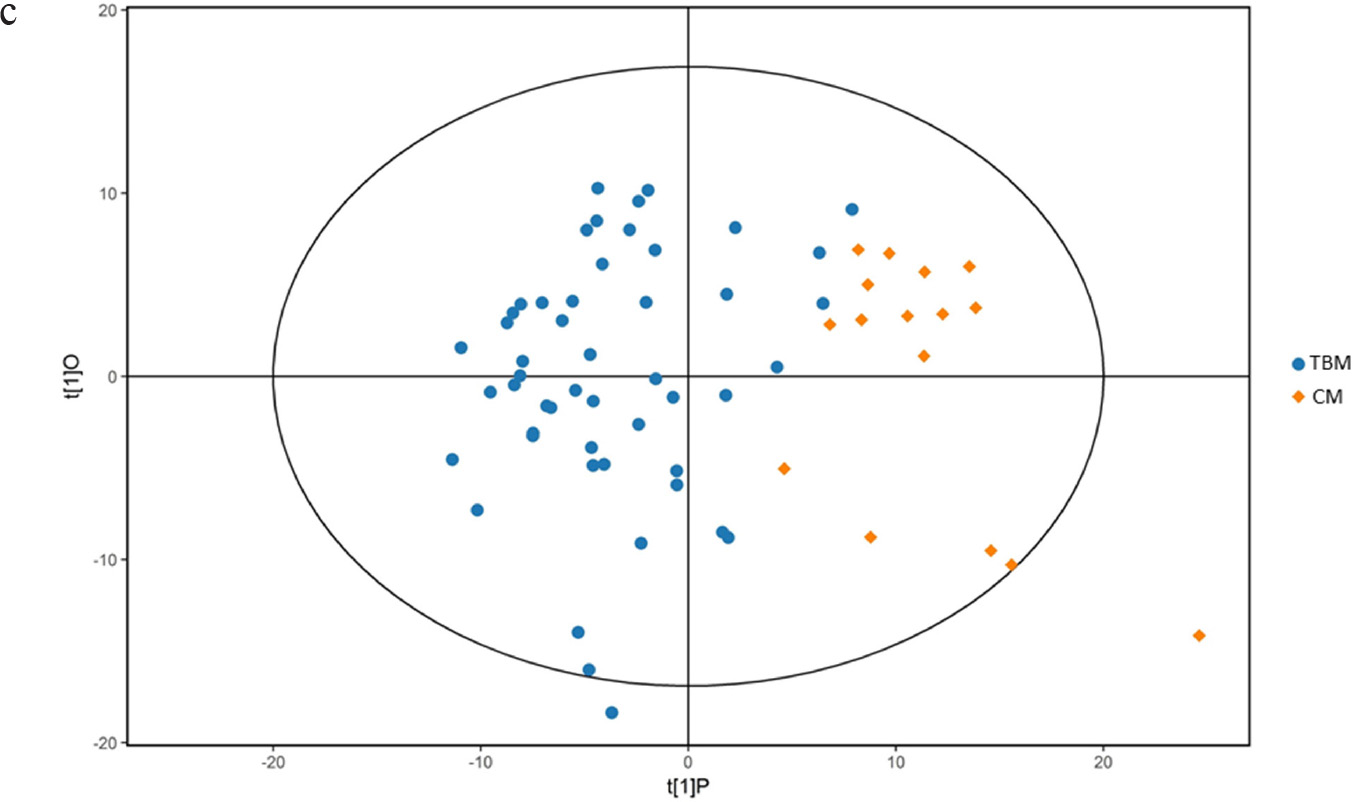

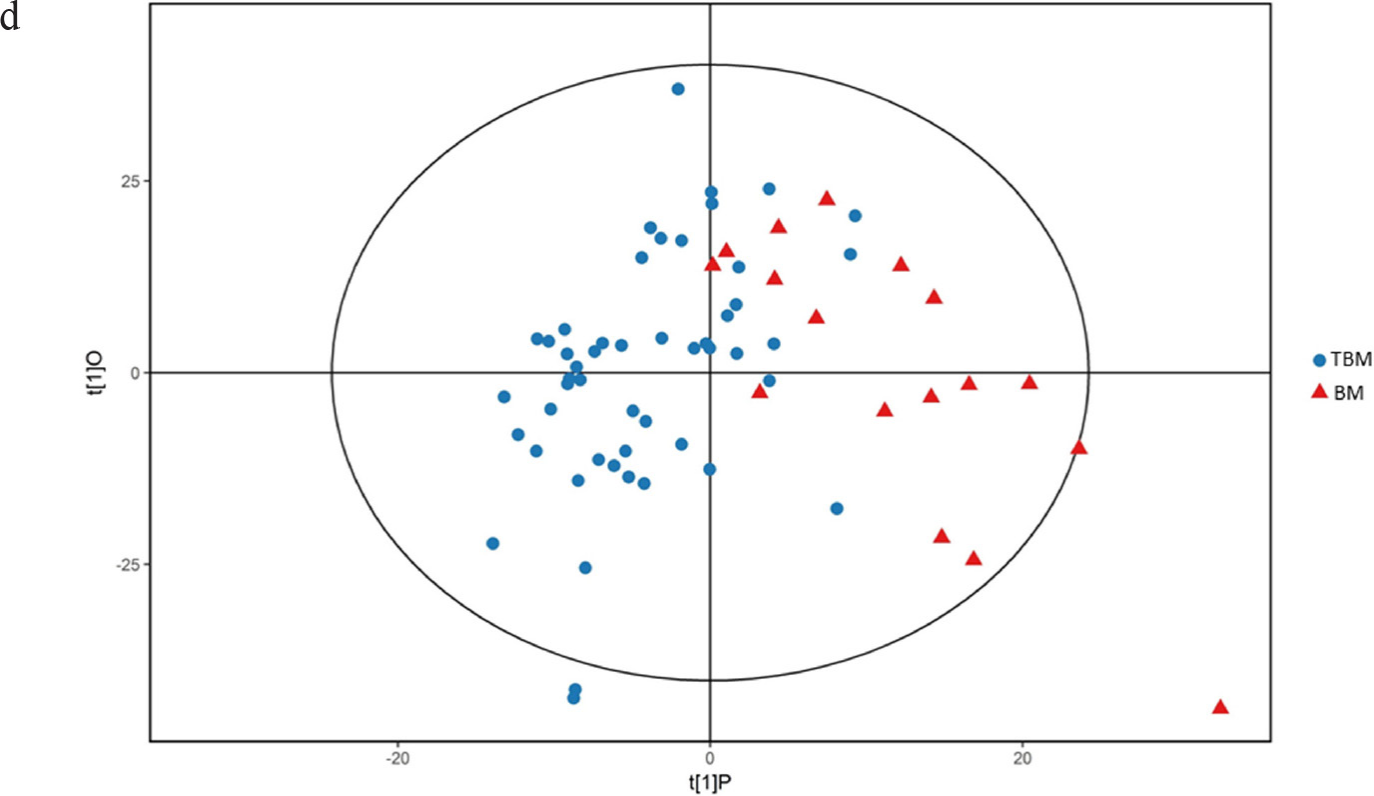

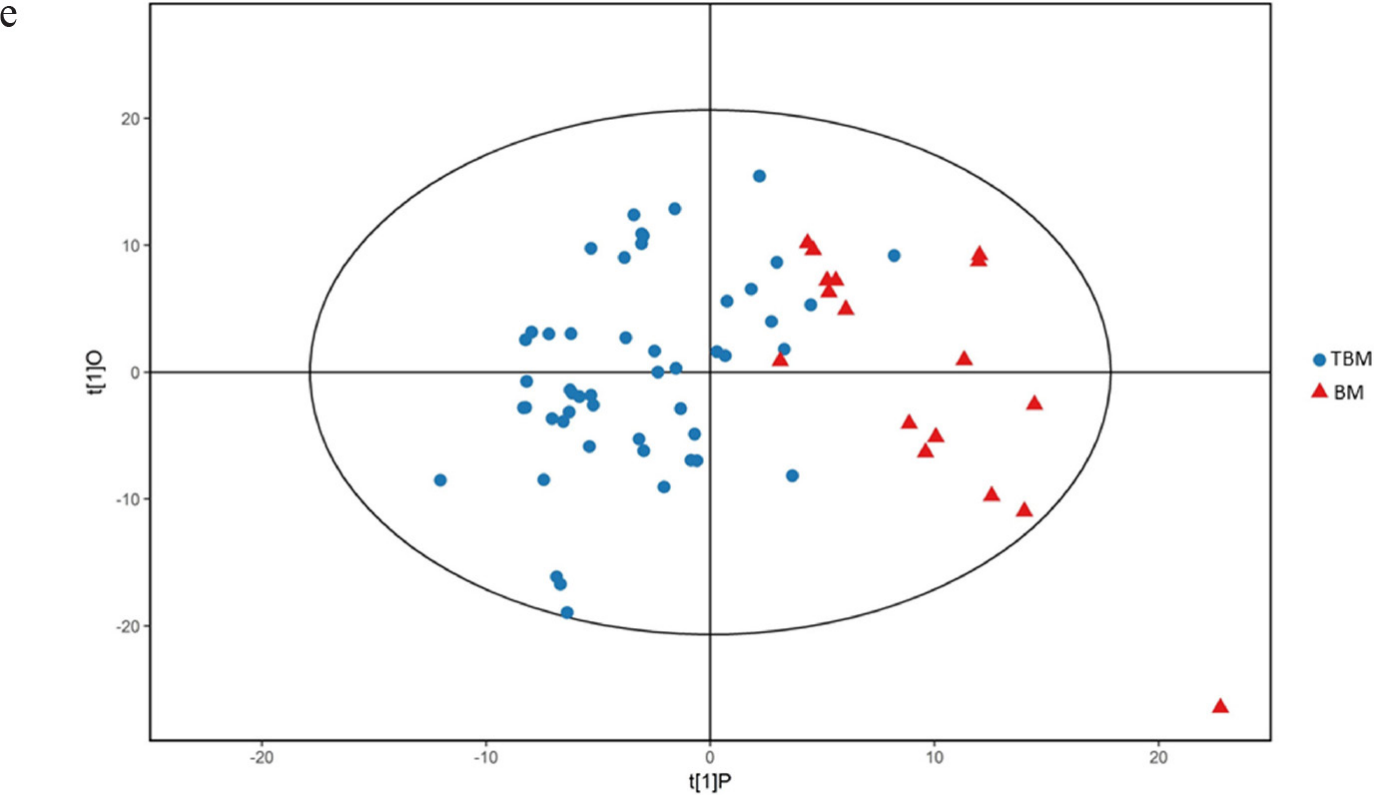

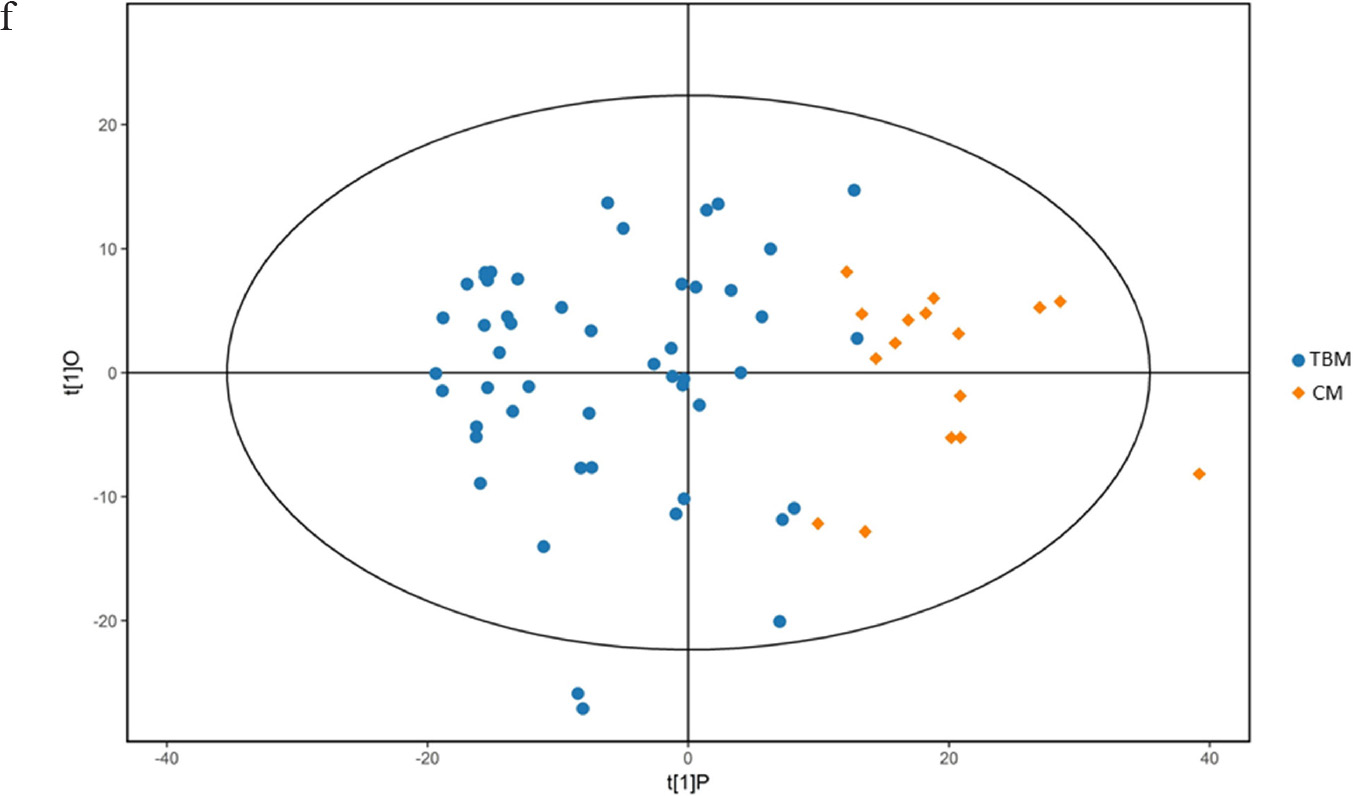
Orthogonal projections to latent structures-discriminate analysis (OPLS-DA) score plots for comparisons between **(a)** Tuberculous meningitis (TBM) and viral meningitis (VM) in positive electrospray ionization (ESI) mode; **(b)** TBM and VM in negative ESI mode; **(c)** TBM and bacterial meningitis (BM) in positive ESI mode. **(d)** TBM and BM in negative ESI mode; **(e)** TBM and cryptococcal meningitis (CM) in positive ESI mode; **(f)** TBM and CM in negative ESI mode. OPLS-DA showed clear discrimination between TBM and viral meningitis, bacterial meningitis, cryptococcal meningitis.

**Figure 3:**
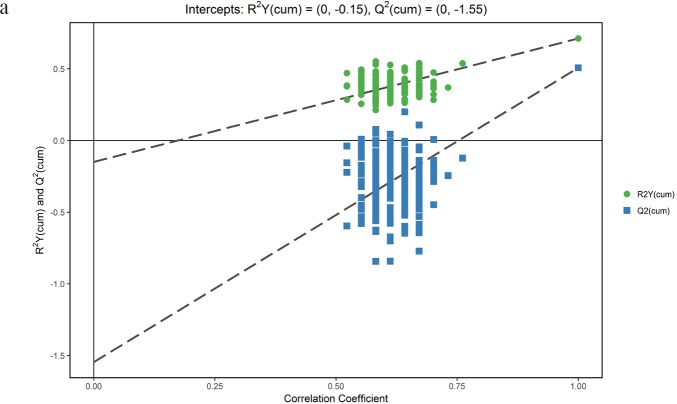

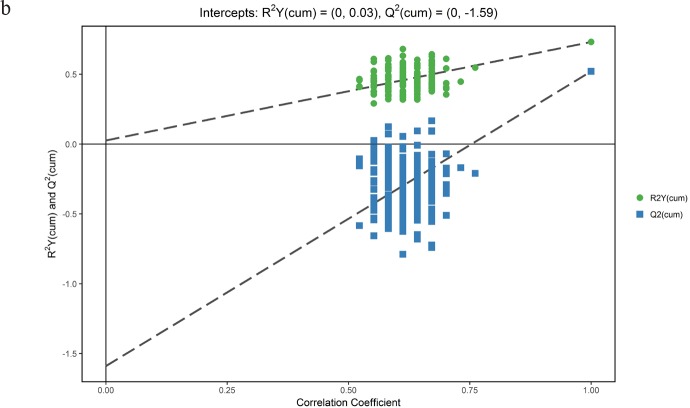

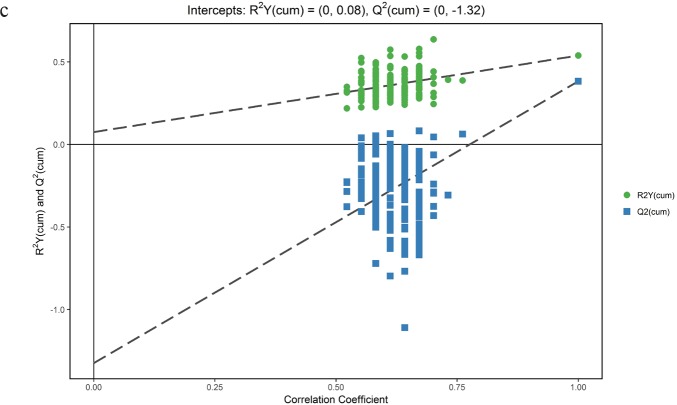

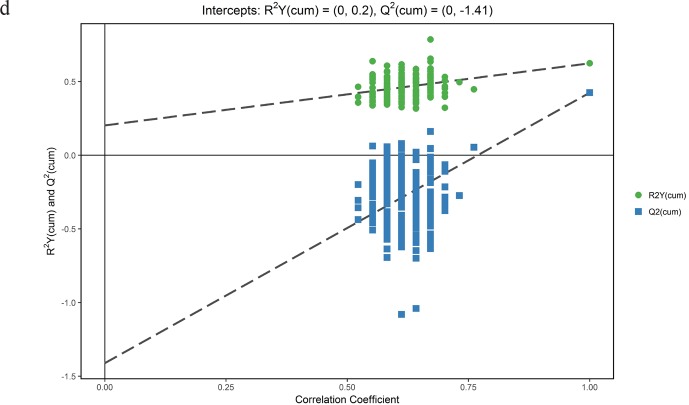

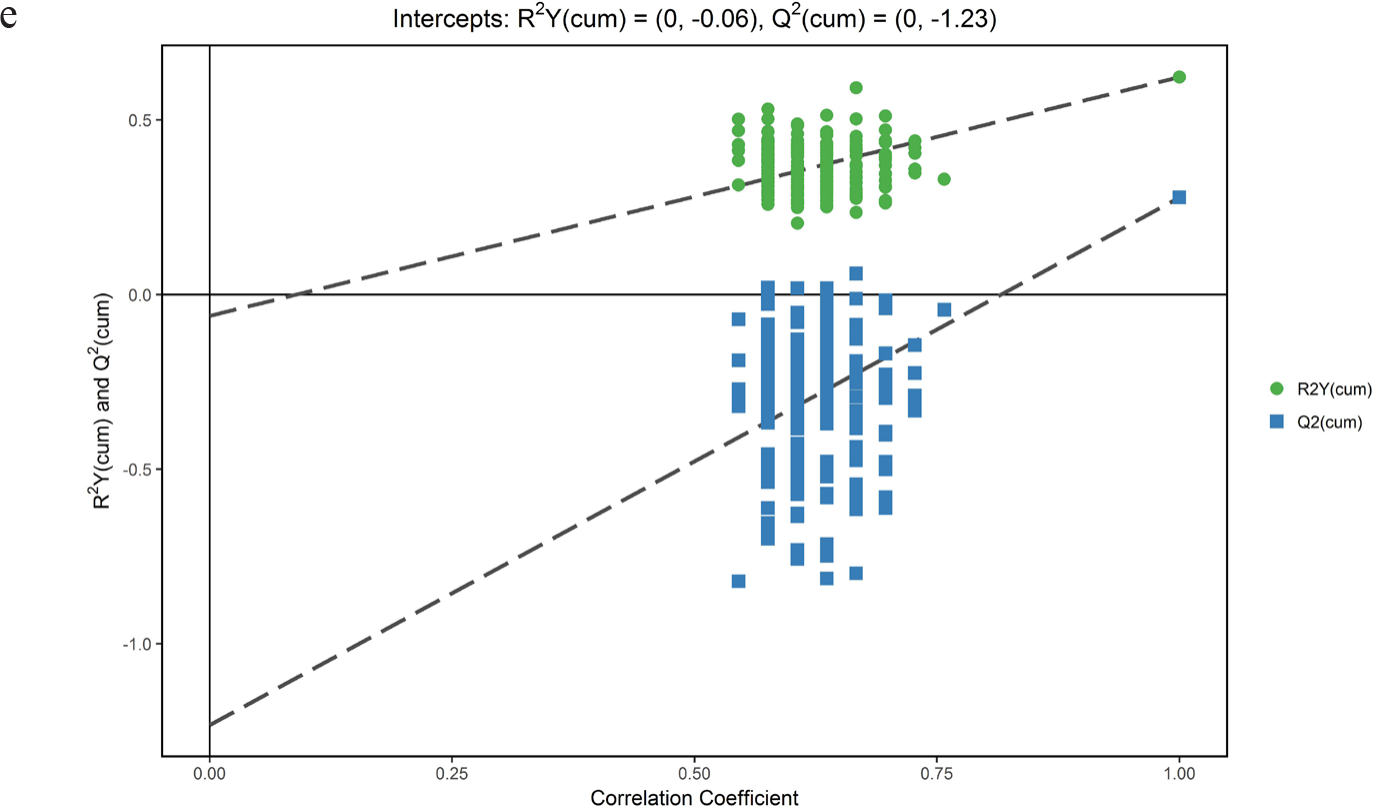

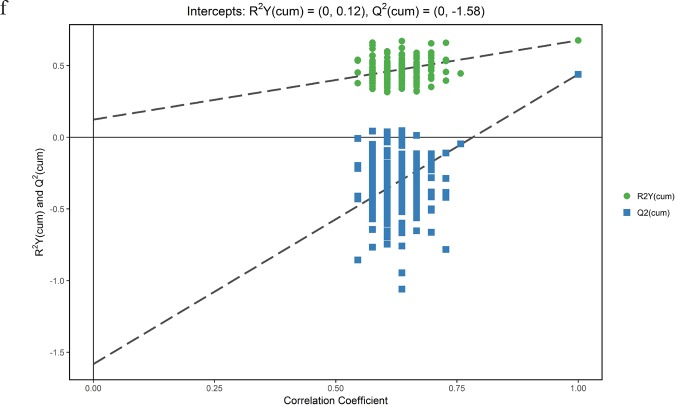
Orthogonal projections to latent structures-discriminate analysis (OPLS-DA) permutation correlation test The horizontal axis indicates the correlation between the ‘real’ and the permuted ‘y’ class. The vertical axis represents R^2^ (goodness-of-fit) and Q^2^ (goodness-of-prediction) values of each model. **(a)** Tuberculous meningitis (TBM) versus viral meningitis (VM) in positive electrospray ionization (ESI) mode; **(b)** TBM versus VM in negative ESI mode; **(c)** TBM versus bacterial meningitis (BM) in positive ESI mode. **(d)** TBM versus BM in negative ESI mode; **(e)** TBM versus cryptococcal meningitis (CM) in positive ESI mode; **(f)** TBM versus CM in negative ESI mode. OPLS-DA permutation correlation test indicated robust models with good fitness and prediction.

We first tried a PCA analysis but we did not find a discernible separation among the four groups (Figure 1). We then tried OPLS-DA and found that it was able to separation TBM from viral meningitis (Figure 2a, 2b), from bacterial meningitis (Figure 2c, 2d), and from cryptococcal meningitis (Figure 2e, 2f). The quality of the assessing models was evaluated by sevenfold cross-validation permutation test (Figure 3). Related parameters were exhibited in Table 1. R^2^X and R^2^Y are the cumulative modeled variation in X and Y matrix, respectively. Q2 is the cumulative predicted variation in Y matrix. Larger R^2^Y and Q^2^ values indicated more robust models with better fitness and prediction. According to our data, the R^2^Y and Q^2^ values in each comparison revealed ideal data models.

**Table 1:** Summary of the parameters of OPLS-DA models

Comparison	R^2^X (cum)	R^2^Y (cum)	Q^2^ (cum)
TBM vs. VM (ESI+)	0.421	0.712	0.507
TBM vs. VM (ESI-)	0.334	0.732	0.521
TBM vs. BM (ESI+)	0.408	0.539	0.383
TBM vs. BM (ESI-)	0.319	0.624	0.425
TBM vs. CM (ESI+)	0.413	0.623	0.278
TBM vs. CM (ESI-)	0.328	0.676	0.439

OPLS-DA: Orthogonal projections to latent structures-discriminate analysis; TBM: Tuberculous meningitis; VM: Viral meningitis; BM: Bacterial meningitis; CM: Cryptococcal meningitis.

R^2^X and R^2^Y are the cumulative modeled variation in X and Y matrix, respectively. Q^2^ is the cumulative predicted variation in Y matrix.

### Potential metabolic markers and related pathways

**Figure 4:**
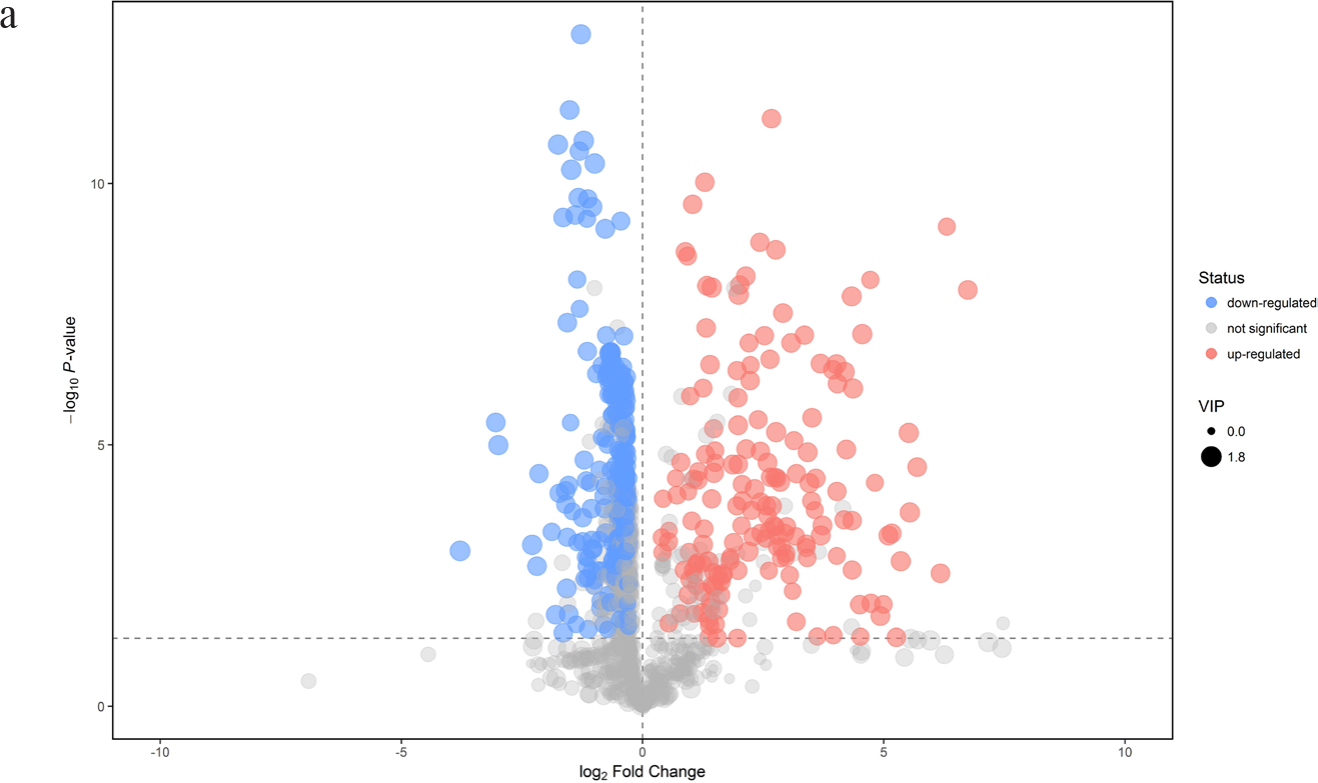

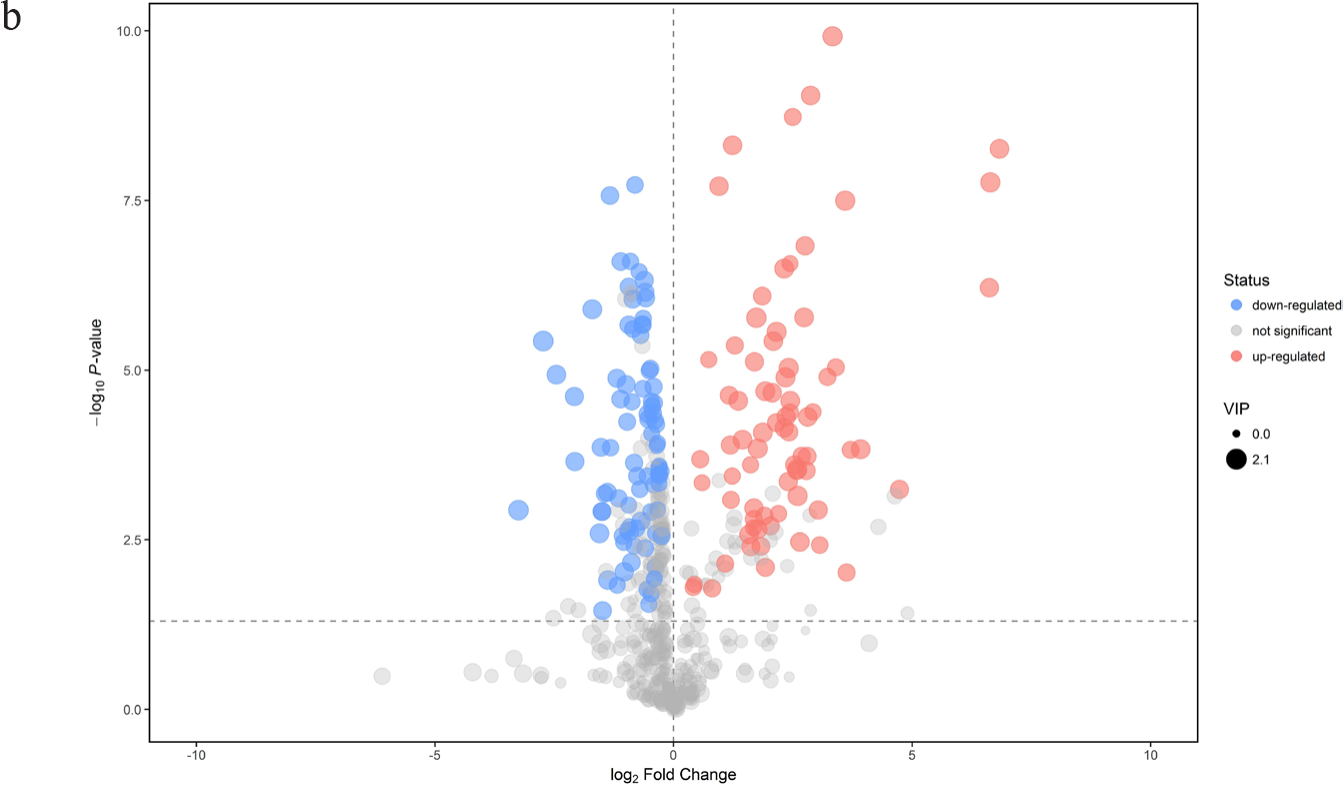

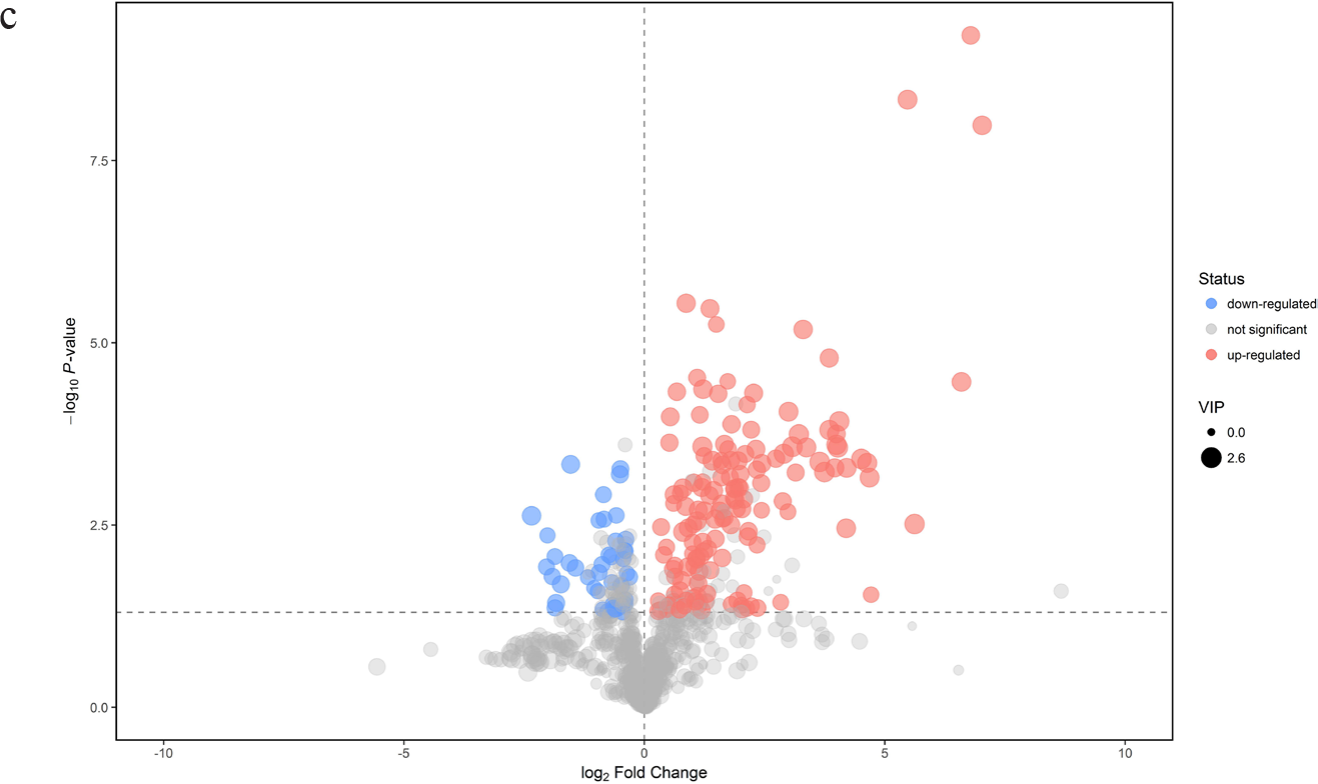

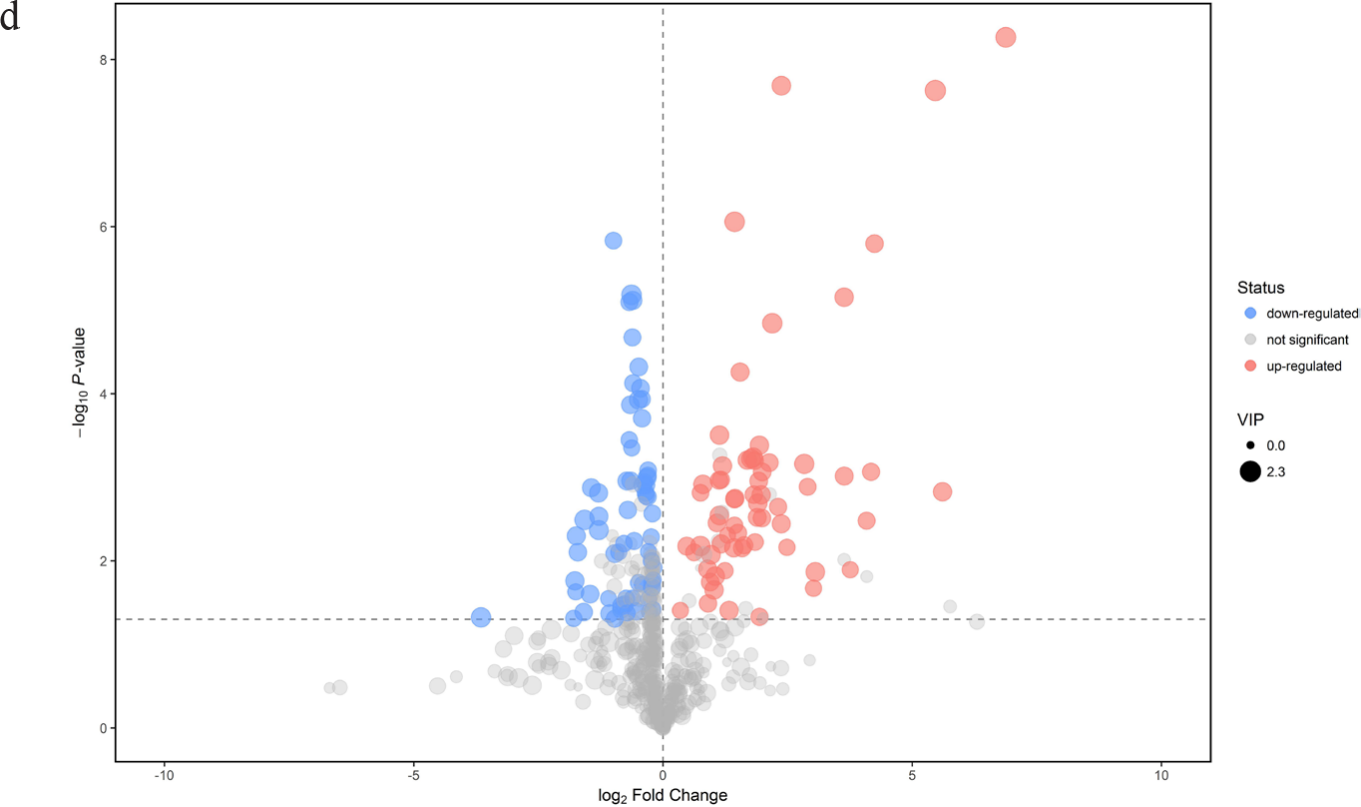

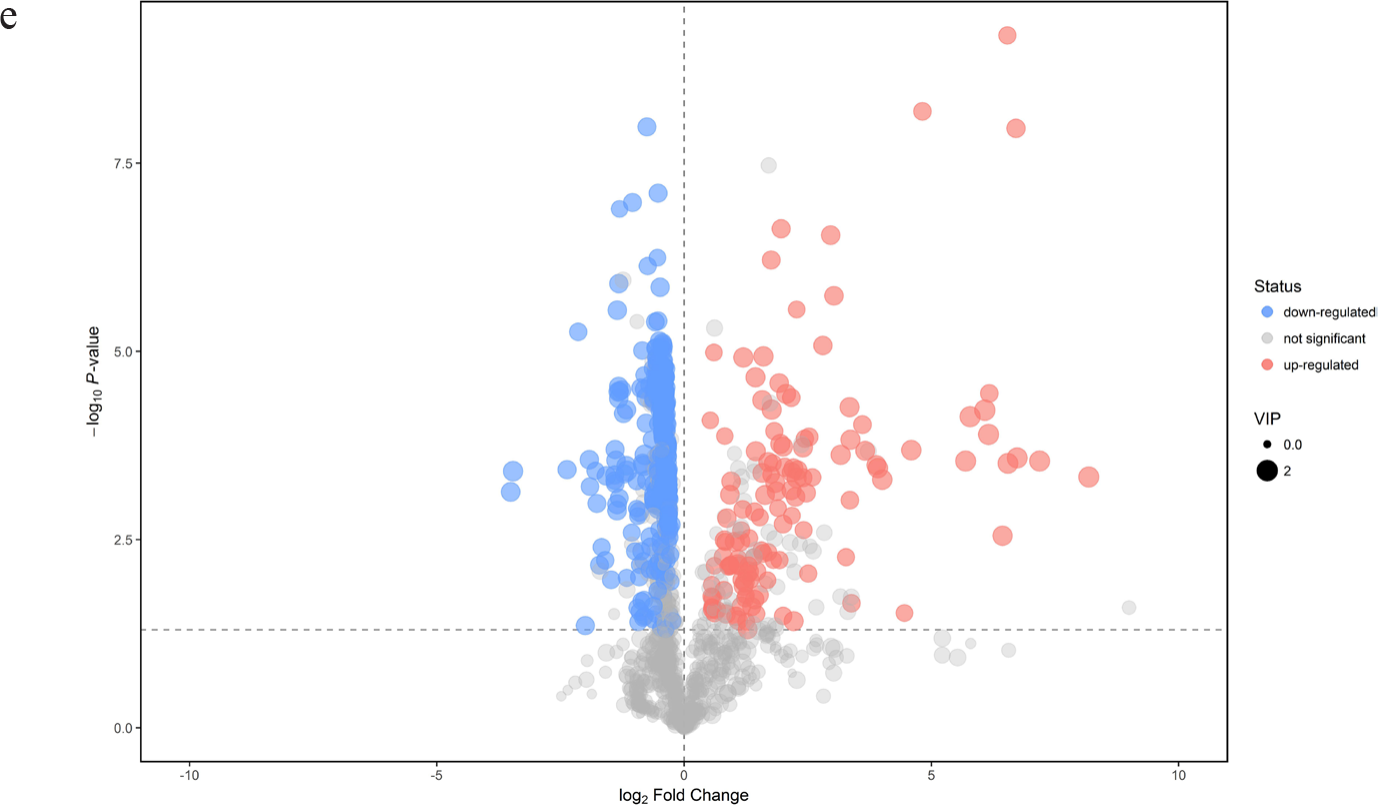

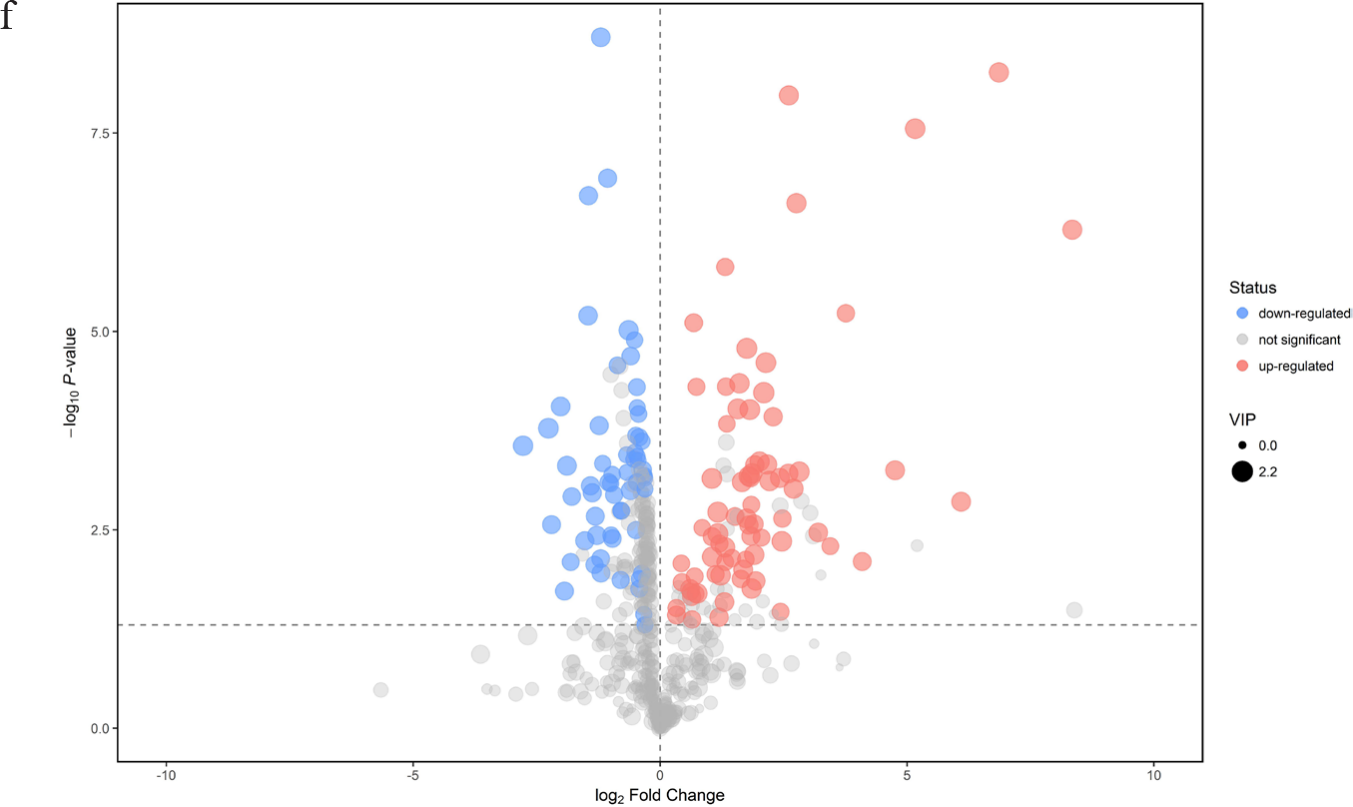
Volcano plots of differentially expressed compounds for each comparison Red spots indicated up-regulated metabolites in TBM, while blue spots demonstrated down-regulated metabolites. Grey spots showed no significant difference in the comparison. The spot size represented the value of variable importance in the projection (VIP). The horizontal axis was related to the value of fold change (FC), while the vertical axis was associated with *P* value. **(a)** Tuberculous meningitis (TBM) versus viral meningitis (VM) in positive electrospray ionization (ESI) mode; **(b)** TBM versus VM in negative ESI mode; **(c)** TBM versus bacterial meningitis (BM) in positive ESI mode. **(d)** TBM versus BM in negative ESI mode; **(e)** TBM versus cryptococcal meningitis (CM) in positive ESI mode; **(f)** TBM versus CM in negative ESI mode.

Visible volcano plots of differentially expressed compounds for each comparison were displayed in Figure 4. Potential metabolic markers which contributed to the discriminative power were selected based on the following criteria. The individual variable importance in the projection (VIP) used in the first principal component of the OPLS-DA model was obtained and initially selected if the value exceeded 1. Secondly, a *P* value of the student t-test for the intergroup comparison should be less than 0.05. Meanwhile, the fold change (FC) larger than 2 or less than 0.5 was also considered as an identification standard. The relative intensities of these metabolites across samples in each group were depicted by heatmap (Supplementary Figures 1-6). We further formulated extra stringent criteria of VIP > 1.5 and *P* value <0.01 to narrow the scope of identified biomarkers to a final list of 13 metabolites in the comparison between TBM and viral meningitis, 16 in TBM and bacterial meningitis and 9 in TBM and cryptococcal meningitis, as was shown in Table 2. Most of the metabolites exhibited increased contents in the CSF of TBM patients compared to other types of meningitis, except for lower 3,4-Dihydroxybenzoate concentration in TBM than viral meningitis, decreased level of Chenodeoxycholate and Phosphatidic acid (PA 18:1/0:0) in TBM than bacterial meningitis.

**Table 2:** Discriminating metabolites in different meningitis

Metabolites^*^	RT (min)	M/Z (Th)	VIP	*P value*	FC
Urea^1a^	140.008	124.050929	1.503891909	1.0856E-08	106.8403516
Uric acid^1a^	59.466	169.0359991	1.766390499	1.33364E-08	3.973818981
Linoleic acid^1a^	449.7595	298.2741351	1.652824232	3.45446E-05	2.781309543
Chenodeoxycholate^1a^	448.1235	410.3261089	1.647384546	8.38041E-07	20.57733569
Stearoylcarnitine^1a^	421.783	428.372987	1.523924897	4.37533E-05	12.029583
PE(P-16:0/0:0)^1a^	428.9775	438.297624	1.572004447	0.000495351	35.9149709
PE(18:0/0:0)^1a^	465.721	482.3249739	1.554053472	0.000543612	34.21219363
Bilirubin^1a^	615.1005	607.2515801	1.51429264	2.65611E-05	51.6479856
3,4-Dihydroxybenzoate^1b^	197.441	153.0181797	1.807539672	1.27153E-06	0.307684576
PA(18:0/0:0)^1b^	443.139	437.2568706	1.90169195	1.20599E-10	10.10066752
Glycocholic acid^1b^	259.83	464.299022	1.621694964	0.000438555	5.290085601
Taurochenodeoxycholate^1b^	287.565	498.2863073	1.767387099	1.66982E-06	6.663921618
PE(22:0/20:4)^1b^	521.438	822.6109011	1.92561168	0.000715407	6.077756893
Urea^2a^	140.008	124.050929	2.134332187	1.04145E-08	130.2227673
N1-Methyl-2-pyridone-5-carboxamide^2a^	138.983	153.06621	1.585814553	3.00975E-05	2.139025595
Uric acid^2a^	59.466	169.0359991	1.937970294	3.39892E-06	2.57269939
Linoleic acid^2a^	411.921	263.2372955	1.778685555	0.001237247	2.552207237
Palmitic acid^2a^	369.4995	279.2333081	1.504569019	0.005486157	2.008442057
cis-9-Palmitoleic acid^2a^	427.993	296.2586104	1.843573474	0.007348918	2.364154318
16-Hydroxypalmitic acid^2a^	369.503	314.2691053	1.765525354	0.001395928	4.190631748
all cis-(6,9,12)-Linolenic acid^2a^	410.488	320.2561186	1.740616894	0.000475345	3.080682499
Lathosterol^2a^	465.016	369.3471161	1.763848931	0.005390979	2.31844807
Erucamide^2a^	603.5535	401.3622489	2.29065995	0.000272141	10.33880254
PC(12:0/26:2)^2a^	594.785	814.6242415	2.255817949	0.000707563	25.67970455
PC(12:0/26:1)^2a^	593.091	816.631561	2.386979876	0.0003332	7.466268588
Stearic acid^2b^	467.586	283.2618291	1.635248205	0.000622651	3.226339677
Chenodeoxycholate^2b^	316.6085	391.2818317	2.006783515	0.003234407	0.335692003
PA(18:1/0:0)^2b^	403.176	435.2488006	1.590664287	0.002926343	0.409774426
PA(18:0/0:0)^2b^	443.139	437.2568706	1.751426983	2.05113E-08	5.181520887
cis-9-Palmitoleic acid^3a^	344.485	296.258459	1.627882472	0.000401883	4.524783391
Linoleic acid^3a^	449.7595	298.2741351	1.708657009	0.003317682	2.193969965
16-Hydroxypalmitic acid^3a^	369.503	314.2691053	1.588932065	0.00085829	4.772449361
all cis-(6,9,12)-Linolenic acid^3a^	410.488	320.2561186	1.694832529	3.69472E-05	4.187537142
Erucamide^3a^	603.5535	401.3622489	1.612374035	0.000209225	12.63995394
PC(12:0/26:2)^3a^	594.785	814.6242415	1.939403109	0.000466084	290.4818789
PA(18:0/0:0)^3b^	443.139	437.2568706	1.812136246	1.05865E-08	6.091769188
Glycocholic acid^3b^	259.83	464.299022	1.917884493	0.006536263	3.745026068
PG(18:1/0:0)^3b^	382.032	509.2854154	1.563889895	0.003802418	3.58301664

^*^1: Tuberculous meningitis versus viral meningitis; 2: Tuberculous meningitis versus bacterial meningitis; 3: Tuberculous meningitis versus cryptococcal meningitis; a: Positive electrospray ionization mode; b: Negative electrospray ionization mode.

RT: Retention time; VIP: Variable importance in the projection; FC: Fold change.

PE: Phosphatidylethanolamine; PA: Phosphatidic acid; PC: Phosphatidylcholine; PG: Phosphatidylglycerol.

**Figure 5:**
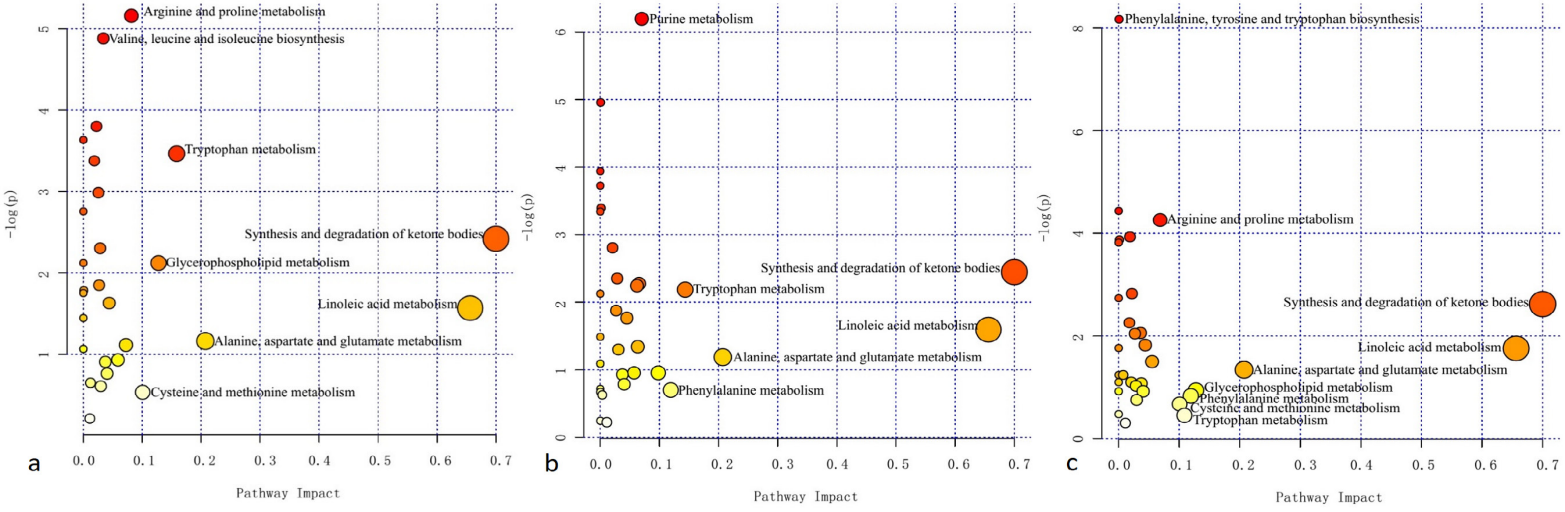
Summary of pathway analysis in each comparison The horizontal axis represented the pathway impact, and the vertical axis was related to the *P* value. **(a)** Tuberculous meningitis (TBM) versus viral meningitis (VM); **(b)** TBM versus bacterial meningitis (BM); **(c)** TBM versus cryptococcal meningitis (CM). Essential pathways with large impacts or significant *P* values were highlighted.

By thoroughly searching the database of KEGG Pathway, the differentially abundant metabolites were cross-referenced to the related pathways. To further learn about the impact value of each pathway, pathway analysis by MetaboAnalyst was processed and presented in Figure 5. Essential pathways with large impacts were labeled in each comparison, with the detailed results of pathway analyses listed in Table 3. In particular, “Total” represented the total number of compounds in the pathway; “Hits” referred to the actually matched number from the user uploaded data; False Discovery Rate (FDR) is the *P* value adjusted using FDR model; the Impact is the pathway impact value calculated from pathway topology analysis. The metabolism of fatty acid and amino acid, e.g. ketone bodies, linoleic acid, alanine, aspartate and glutamate, et al, seemed to participate in the comparisons.

**Table 3:** Results of pathway analyses of the essential pathways

Pathway name^*^	Total^1^	Hits^2^	Raw *P*	FDR^3^	Impact
Synthesis and degradation of ketone bodies^a^	6	1	0.088847	0.78975	0.7
Linoleic acid metabolism^a^	15	1	0.20789	0.97829	0.65625
Alanine, aspartate and glutamate metabolism^a^	24	1	0.31174	1	0.20703
Tryptophan metabolism^a^	79	4	0.031215	0.45459	0.15839
Glycerophospholipid metabolism^a^	39	2	0.11957	0.79711	0.12753
Cysteine and methionine metabolism^a^	56	1	0.58424	1	0.10058
Arginine and proline metabolism^a^	77	5	0.0057482	0.30356	0.08151
Valine, leucine and isoleucine biosynthesis^a^	27	3	0.0075889	0.30356	0.03392
Purine metabolism^b^	92	6	0.0020401	0.16321	0.07
Synthesis and degradation of ketone bodies^b^	6	1	0.086536	0.73324	0.7
Tryptophan metabolism^b^	79	3	0.11219	0.73324	0.14349
Linoleic acid metabolism^b^	15	1	0.20284	1	0.65625
Alanine, aspartate and glutamate metabolism^b^	24	1	0.3047	1	0.20703
Phenylalanine metabolism^b^	45	1	0.49563	1	0.11906
Phenylalanine, tyrosine and tryptophan biosynthesis^c^	27	4	0.00028228	0.022583	0.00062
Arginine and proline metabolism^c^	77	4	0.014107	0.29182	0.06844
Synthesis and degradation of ketone bodies^c^	6	1	0.072563	0.64501	0.7
Linoleic acid metabolism^c^	15	1	0.17195	0.91707	0.65625
Alanine, aspartate and glutamate metabolism^c^	24	1	0.261	1	0.20703
Glycerophospholipid metabolism^c^	39	1	0.38925	1	0.12753
Phenylalanine metabolism^c^	45	1	0.43427	1	0.11906
Cysteine and methionine metabolism^c^	56	1	0.50863	1	0.10058
Tryptophan metabolism^c^	79	1	0.63481	1	0.10853

^*^a: Tuberculous meningitis versus viral meningitis; b: Tuberculous meningitis versus bacterial meningitis; c: Tuberculous meningitis versus cryptococcal meningitis.

^1^ Total: total number of compounds in the pathway; ^2^ Hits: the number of compounds that match with our experimental data;^ 3^ FDR: False Discovery Rate.

## DISCUSSION

With the development of drug-resistance, TB still remains a serious health burden all over the world at the present time [15]. According to the Disability-adjusted life years (DALYs) proposed by the 2010 Global Burden of Disease Study, TB is 13th cause of disability, and the 10th factor of death [16]. TBM presents with disseminated TB infection with CNS involvement, and has been recognized as a critical form of TB. However, the diagnosis of TBM is often obscured due to its non-specific clinical appearance. Furthermore, the differential diagnosis of TBM with viral meningitis, bacterial meningitis and cryptococcal meningitis is a tough challenge in some cases. In this study, we conduct a metabolomics study to investigate the metabolic profiles of the CSFs of TBM and other types of meningitis, and to identify potential biomarkers to differentiate TBM from other types of meningitis.

Intriguingly, we found an increase in the levels of a large amount of metabolites in TBM, as compared with other types of meningitis. Six major groups of altered metabolites, including amino acids, urea, lipids, fatty acids, bilirubin, bile acid, and uric acid, were found to to be different from the comparisons. These substances mainly participated in the metabolism of amino acids (diverse amino acids including leucine, proline, valine, phenylalanine, glutamine, tyrosine, tryptophan, homocysteine, and urea), lipids (lathosterol, PA, phosphatidylethanolamine (PE), phosphatidylcholine (PC), phosphatidylglycerol (PG); fatty acids including linoleic acid, palmitic acid, 16-hydroxypalmitic acid, stearic acid; bilirubin; bile acids such as chenodeoxycholate, 3,4-dihydroxybenzoate, glycocholic acid; and other metabolite of lipid like stearoylcarnitine), and nucleoside (uric acid, xanthine, hypoxanthine and urea), et al.

TB infection requires sufficient energy supply, which leads to the altered concentrations of a variety of energy-associated metabolites in the CSF. Amino acids take part either directly or indirectly in a diversity of essential biochemical functions in CNS, including the provision of building blocks for energy production through gluconeogenesis, the intercellular shuttles between mitochondria, and mitochondrial ATP production [13, 17]. In this study, various amino acids presented with an elevated amount in the CSF of TBM (Table 2). In addition, several related pathways in the three groups of comparisons were closely associated with the metabolism of amino acids. On the other hand, urea is involved in the metabolism of arginine and proline. The increased level of urea in TBM compared to either viral meningitis, bacterial meningitis or cryptococcal meningitis might also be related to vigorous amino acid metabolism.

As an important enzyme involved in nucleic acid metabolism, adenosine deaminase (ADA) is associated with the activity of cellular immunity. Reported data showed that ADA could be used as a biomarker in the diagnosis of TBM [18]. Previous studies have found purine metabolism related-molecules, such as xanthine, urate, malonate and malonic acid, increased in TBM, rather than other types of meningitis [19, 20]. We have demonstrated that uric acid is significantly higher in the CSF of TBM than viral or bacterial meningitis. Besides, the level of urea, xanthine and hypoxanthine also presented with the same trend. Furthermore, purine metabolism was proved to be a related pathway in the comparison of TBM with bacterial meningitis. These results have indicated TBM might alter nucleoside metabolism, and related markers could serve as potential candidates for meningitis differentiation.

In this study, we found that numerous unsaturated fatty acids present with an elevated level in TBM. Moreover, altered lipid and bile acid metabolism was observed. In TBM, an increased synthesis and degradation of ketone bodies resulted in an elevated concentration of acetoacetic acid. These phenomena may be due to enhanced glycerolipid and glycerophospholipid metabolism to meet the increased energy requirement in TBM. Nevertheless, how lipid metabolism is modified in the complex pathogenic process warrants further research.

This study is one of the first metabolomics studies for CSFs of TBM, viral meningitis, bacterial meningitis and cryptococcal meningitis. Abundant metabolites were identified to discriminate TBM and other types of meningitis, and could be used in the diagnosis of TBM. The involvement of some substances has never been reported in published literatures. However, certain limitations of this study should be taken into consideration. Firstly, some differentially expressed molecules with certain m/z ratios and RTs were recognized as unknown substances due to lack of database. Secondly, targeted metabolomics analyses for the validation of potential biomarkers were not performed, due to limited samples. Future studies are necessary to validate the findings and to analyze the links between the altered metabolites and pathophysiology of diseases.

In conclusion, metabolomics analyses based on UHPLC-QTOF-MS demonstrated altered metabolic profiles in TBM, viral meningitis, bacterial meningitis and cryptococcal meningitis. Related metabolites and pathways are involved in the metabolism of amino acid, lipids and nucleosides. The identification of biomarkers by metabolomics in CSF provides valuable information for the diagnosis and differential diagnosis of TBM, and may contribute to the reduction of TBM morbidity and mortality.

## MATERIALS AND METHODS

### Patients and CSF samples

Patients with TBM, viral meningitis, bacterial meningitis and cryptococcal meningitis in the Department of infectious diseases, Zhejiang Provincial People’s Hospital were consecutively enrolled from January, 2014 to December, 2015, with CSF samples collected. The diagnosis of various types of meningitis was based on the procedure of authoritative guidelines [21–24]. All patients should have symptoms and clinical signs of meningitis. TBM: recent exposure to tuberculosis, typical changes of CSF, including protein >1 g/L, pleocytosis (>20 cells/μL), lymphocytes >60%, and CSF to blood glucose ratio of <0.6, with or without acid-fast bacilli seen or cultured in the CSF. Patients with TBM had response to anti-tuberculosis therapy. Viral meningitis: an elevated white blood cell count (usually 10-100 cells/μL) with a lymphocytic predominance and a normal glucose level. Bacterial meningitis: presence of white blood cells, red blood cells, elevated protein content and glucose level in CSF samples. Cryptococcal meningitis: positive India ink stains of CSF for Cryptococcus.

Children and adolescents whose ages were less than 18 years should be excluded. Patients with human immunodeficiency virus (HIV) infection were also excluded. The study was processed in agreement with the declaration of Helsinki, and was approved by the ethnic committee of Zhejiang Provincial People’s Hospital. All subjects were informed about the objective and design of study, and have signed a written informed consent. A lumbar CSF sample was taken upon admission before any treatment in each patient, and was stored at -80°C until further analysis. We intend to compare the CSF metabolic variations of TBM with viral meningitis, bacterial meningitis and cryptococcal meningitis, respectively.

### Sample preparation

Samples were thawed at room temperature and 150μL of CSF was taken into the 1.5 mL EP tubes. 300μL methanol was added, followed by 20μL L-2-Chlorophenylalanineas (1 mg/mL stock in dH_2_O) as an internal standard to the sample. The mixture was homogenized using vortex for 30s, and sonicated for 10min (incubated in ice water). After incubating at -20°C for 1 hour, CSF samples were centrifuged at 12000rpm, 4°C for 15 min. 0.2mL of the supernatant was transferred into a 2ml LC/MS glass vial. 10μL of each sample was taken and pooled as quality control (QC) samples.

### UHPLC-QTOF-MS analysis

Samples were analyzed by using Agilent 1290 UHPLC system (Agilent 1290, Agilent, USA), coupled with Agilent 6550 QTOF mass spectrometer (Agilent 6550, Agilent, USA) and AB Triple TOF 6600 system (AB 6600, SCIEX, USA). ACQUITY UPLC HSS T3 system (Waters, Milford, MA) was utilized with a 2.1 × 100 mm, 1.7μm C18 column maintained at 40°C for chromatographic analysis. All samples were analyzed in both positive and negative electrospray ionization (ESI+/-) modes. The mobile phase for positive ESI mode consists of (A) 0.1% formic acid (FA) in water and (B) 0.1% FA in acetonitrile (ACN). For negative ESI mode, it was composed of (A) 0.5mM ammonium fluoride in water and (B) ACN. The flow rate was set at 500μl/min, with the gradient optimized as follows (time (minute), A %): (0, 99), (1, 99), (8, 0), (10, 0), (10.1, 99), (12, 99).

### Data processing and statistical analyses

The raw data were processed by XCMS software 1.41.0 for peaks extracting, filtrating and matching [25]. All peaks were defined by m/z ratios and retention times (RT). The matching standard was detailed as “m/z tolerance ± 30ppm” and “RT tolerance ± 60s”. Interquartile range de-noising method and overall normalization by support vector regression (SVR) method was employed, while missing data were filled up by half of the minimum value.

The resulted data were fed to SIMCA software (Version 14.1, MKS Data Analytics Solutions, Umea, Sweden) for principal component analysis (PCA) and orthogonal projections to latent structures-discriminate analysis (OPLS-DA). PCA demonstrated the distribution of original data; while OPLS-DA was applied for a better observation of group separation. 7-fold cross validation was applied to validate the robustness and the predictive ability of our model. Furthermore, commercial databases including Kyoto Encyclopedia of Genes and Genomes (KEGG) (http://www.genome.jp/kegg/) and MetaboAnalyst (http://www.metaboanalyst.ca/) was consumed to search for the pathways of metabolites.

## SUPPLEMENTARY MATERIALS FIGURES


