# Supplementary material for: Identification of potential metabolic biomarkers of cerebrospinal fluids that differentiate tuberculous meningitis from other types of meningitis by a metabolomics study
Source: Oncotarget. 2017 Oct 19;8(59):100095–112. doi: 10.18632/oncotarget.21942 (PMC5725005; doi:10.18632/oncotarget.21942)
Supplement: Supplementary file 1 [file oncotarget-08-100095-s001.pdf]

Identification of potential metabolic biomarkers of cerebrospinal fluids that differentiate tuberculous meningitis from other types of meningitis by a metabolomics study

SUPPLEMENTARY MATERIALS

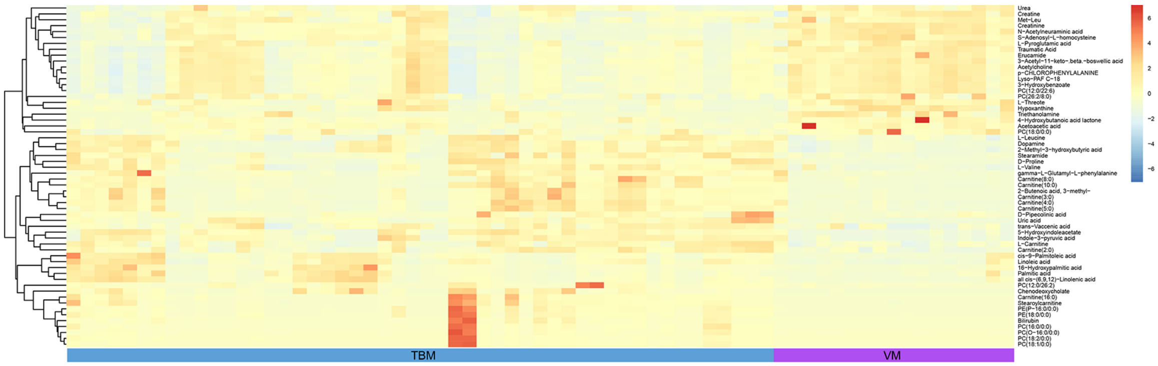

Supplementary Figure 1: Heatmap of discriminating metabolites in the comparison of Tuberculous meningitis versus viral meningitis in positive electrospray ionization mode. Columns referred to individual samples and rows represented distinct metabolites. Shades of red or blue represented the elevation or decrease of a metabolite in TBM, respectively.

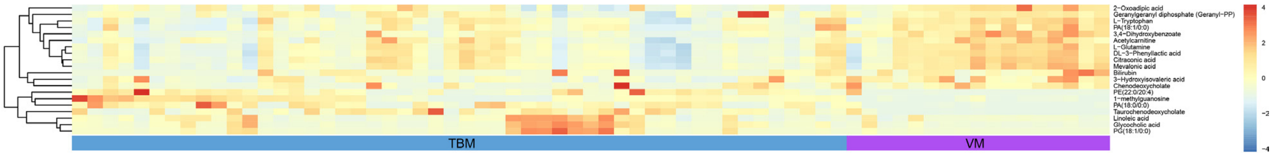

Supplementary Figure 2: Heatmap of discriminating metabolites in the comparison of Tuberculous meningitis versus viral meningitis in negative electrospray ionization mode. Columns referred to individual samples and rows represented distinct metabolites. Shades of red or blue represented the elevation or decrease of a metabolite in TBM, respectively.

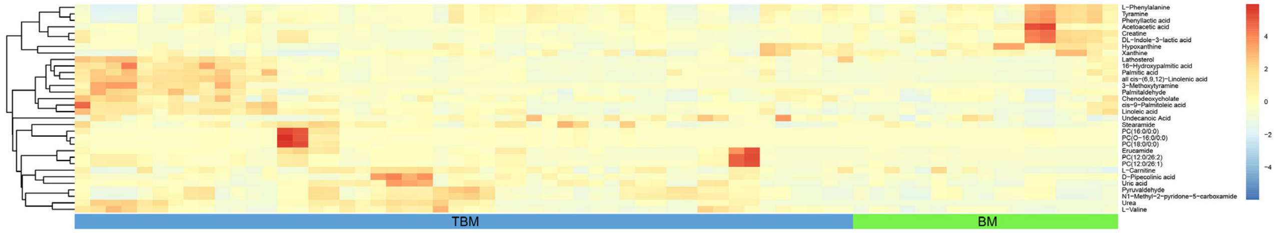

Supplementary Figure 3: Heatmap of discriminating metabolites in the comparison of Tuberculous meningitis versus bacterial meningitis in positive electrospray ionization mode. Columns referred to individual samples and rows represented distinct metabolites. Shades of red or blue represented the elevation or decrease of a metabolite in TBM, respectively.

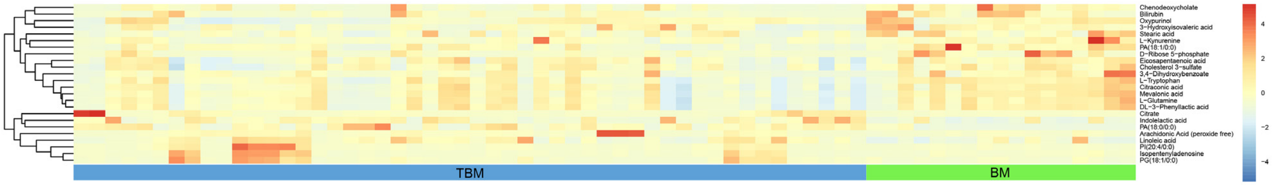

Supplementary Figure 4: Heatmap of discriminating metabolites in the comparison of Tuberculous meningitis versus bacterial meningitis in negative electrospray ionization mode. Columns referred to individual samples and rows represented distinct metabolites. Shades of red or blue represented the elevation or decrease of a metabolite in TBM, respectively.

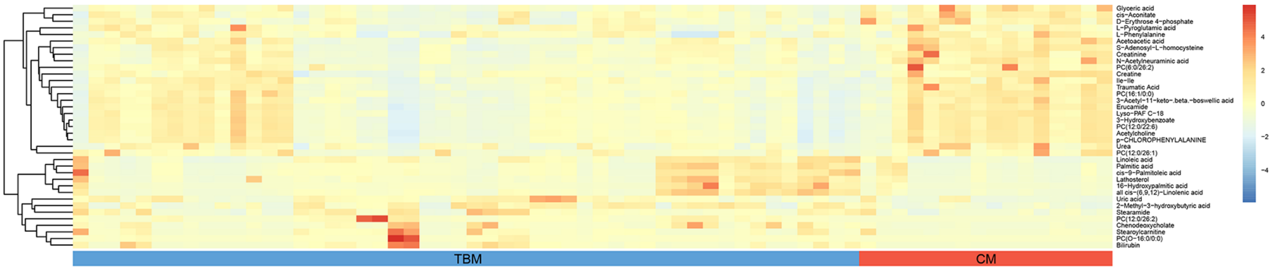

Supplementary Figure 5: Heatmap of discriminating metabolites in the comparison of Tuberculous meningitis versus cryptococcal meningitis in positive electrospray ionization mode. Columns referred to individual samples and rows represented distinct metabolites. Shades of red or blue represented the elevation or decrease of a metabolite in TBM, respectively.

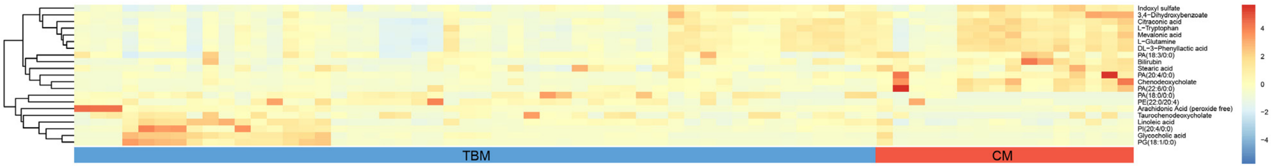

Supplementary Figure 6: Heatmap of discriminating metabolites in the comparison of Tuberculous meningitis versus cryptococcal meningitis in negative electrospray ionization mode. Columns referred to individual samples and rows represented distinct metabolites. Shades of red or blue represented the elevation or decrease of a metabolite in TBM, respectively.
